# Quantitative risk assessment of BSE transmission if insects are reared on residual streams containing animal proteins

**DOI:** 10.3389/fmicb.2026.1943428

**Published:** 2026-10-23

**Authors:** C. J. de Vos, A. F. G. Antonis, M. J. Appel, E. F. Hoek-van den Hil

**Affiliations:** 1 Wageningen Bioveterinary Research, Wageningen University & Research, Lelystad, Netherlands; 2 Wageningen Food Safety Research, Wageningen University & Research, Wageningen, Netherlands

**Keywords:** black soldier fly larvae, bovine spongiform encephalopathy, circularity, exposure assessment, infection probability, waste streams, yellow mealworm

## Abstract

Insect production can play an important role in the bioconversion of residual streams from agri-food production into valuable nutrients for feed and food and as such advance the transition from a linear to a circular food production system. However, under current EU legislation, insect rearing on residual streams containing animal proteins is not allowed because of the risk of animal disease transmission including bovine spongiform encephalopathy (BSE). In this study, a quantitative risk model was used to assess the BSE risk. The model estimates the expected exposure of cattle to BSE infectivity, expressed in cattle oral ID_50_ (CoID_50_), and the corresponding probability that this will result in at least one new BSE infection when a single BSE-infected cow escapes detection. The model was parameterized for three residual streams: former food from supermarkets (SM), kitchen food scraps from consumer households (KF), and category 2 meat meal from rendering (MM); and two insect species: yellow mealworm (*Tenebrio molitor*) (YMW) and black soldier fly (*Hermetia illucens*) larvae (BSFL). Exposure routes considered were cross-contamination of ruminant feed, grassland fertilized with pig or poultry manure or frass, and calf milk replacer. The main uncertainty in the model was the carry-over of prions from substrate to insects. A worst-case approach was used, assuming no inactivation of prions during insect rearing. Similar conservative estimates were used for other parameters describing presence and persistence of prions when no data were available. Model results indicate that the BSE infectivity to which cattle are exposed depends both on the substrate and the insect species, but was very low for all combinations assessed with a maximum median overall exposure of 5.2 × 10^−6^ CoID_50_ for YMW reared on KF, corresponding to a probability of at least one new BSE infection of 3.6 × 10^−6^, implying that < 1 out of 100,000 slaughtered BSE-infected cows will result in new BSE infections. We therefore conclude that insect rearing on residual streams is unlikely to result in the emergence of a new BSE epidemic.

## 1 Introduction

Increasing global demand for food, limited availability of natural resources and environmental side-effects of food and feed production warrant the transition from a linear to a circular food production system in which by-products and waste streams are fed back into the system (Muscat et al., 2021; Doughty et al., 2024). Waste and by-products in the agri-food production chain can either originate at harvest, during processing and manufacturing or at the consumer phase. The United Nations Food Waste Index report estimated that at the consumer phase alone (i.e., retail, food service and households) 19% of total global food production is wasted (UNEP, 2024). Doughty et al. (2024) estimate that the current linear food production system results in a total of 40% of the global food supply being lost or wasted throughout the whole production chain. Insects can play an important role in a circular food production system as bioconvertors of waste and by-products from the food production chain into valuable nutrients for food and feed (Ojha et al., 2020; Doughty et al., 2024; Bruno et al., 2025). Several studies have been undertaken to assess the growth performance of insects on different organic residual streams demonstrating that insect-mediated bioconversion of waste and by-products can contribute to the upcycling of low-value side streams into valuable biomass and nutrients (Broeckx et al., 2021; Naser El Deen et al., 2023; Bruno et al., 2025). The use of waste and by-products as a substrate for insect rearing might, however, entail safety issues, if contaminated with heavy metals, pesticides, veterinary drugs or other chemicals, or pathogenic agents. Studies that evaluated the chemical safety of a variety of former food products and waste streams concluded that most substrates did not present major safety concerns (Van der Fels-Klerx et al., 2020; Meyer et al., 2021; Hoek-van den Hil et al., 2024; Hoffmans et al., 2024; Meijer et al., 2026). Little is known about the microbiological risks of using waste and byproducts as a substrate for insect rearing, and most studies have focused on bacterial contaminations only (Wynants et al., 2019; Vandeweyer et al., 2021; Hoek-van den Hil et al., 2024; Meijer et al., 2026).

Current European Union (EU) legislation restricts the use of waste and by-products containing animal proteins as a source of feed for farmed animals, including insects, because of the risk of disease transmission (EC, 2001, 2009). Feeding of swill (kitchen leftovers and catering waste) has resulted in serious epidemics of contagious animal diseases, including the foot-and-mouth disease epidemic in the United Kingdom in 2001 (Davies, 2002), as well as the current African swine fever epidemic that has affected many countries worldwide (Beltran-Alcrudo et al., 2019; Dixon et al., 2019). Similarly, the wide-spread practice of recycling animal proteins into feed for ruminants resulted in a major epidemic of bovine spongiform encephalopathy (BSE) at the end of the twetieth century. BSE is caused by a misfolded prion protein (PrP^SC^) that is extremely heat resistant and was not inactivated in the default rendering processes applied at that time (Schreuder et al., 1998; Taylor and Woodgate, 2003). A ban on feeding ruminant proteins in ruminant feed was installed in 1994 to stop transmission of BSE, and in 2001 this was extended to a total ban on feeding processed animal proteins to livestock (EC, 2001; Ducrot et al., 2010). These bans resulted in a rapid decline of BSE cases in Europe and almost all EU member states now have a negligible risk status for BSE (Arnold et al., 2017; EFSA, 2018; WOAH, 2024a). Only few cases of BSE have been reported in the EU and the United Kingdom over the last decade, most of which were caused by the so-called atypical BSE strains rather than the classical strain that caused the BSE epidemic (EFSA, 2024a). These atypical BSE types are called H-BSE and L-BSE, because of the respectively higher and lower molecular mass of PrP^SC^ in the Western blot analysis if compared to the classical BSE type (C-BSE) (Alarcon et al., 2023). Atypical BSE occurs sporadically and has only been identified in older cattle (WOAH, 2024a). The origin of the atypical BSE types is unknown, and its risk factors are poorly understood.

In recent years, several relaxations of the total feed ban have been installed including the use of fishmeal in calf milk replacer (EC, 2008), the use of insect meal in aquafeed and pig and poultry feed (EC, 2017, 2021b), and the use of processed animal proteins (PAPs) of poultry origin in pig feed and PAPs of porcine origin in poultry feed (EC, 2021b). Use of ruminant PAPs in feed for non-ruminants remains, however, strictly prohibited because of the potential BSE risk (Meijer et al., 2023). Furthermore, no PAPs of any species can be used as a substrate for insect rearing due to concerns about carry-over of animal proteins from substrate via insects to target animals, which might result in a breach of the species-to-species ban. This limits the use of waste and by-products containing mixed animal proteins as a substrate for insect rearing, such as industrial food waste or catering and kitchen waste, and therewith the advancement of the transition from a linear to a circular food production system.

A better insight in the risk of new BSE infections when rearing insects on residual streams containing animal proteins is needed to make a balanced trade-off between the benefits, i.e., increased circularity, and risks, i.e. food and feed safety of using residual streams. The European Food Safety Authority (EFSA) qualitatively assessed the risk of prion proteins in insect substrates using comparative risk profiles and concluded that the total prion infectivity in insects would be equal or smaller than the amount of infectivity present in the substrate (EFSA, 2015). Similarly, the Netherlands Food and Consumer Product Safety qualitatively assessed the risks of rearing insects on former foodstuffs and identified transfer of prions as a hazard if ruminant proteins would be present (Anonymous, 2019). These studies did, however, not give an indication of the level of risk. The objective of this study was therefore to quantitatively assess the BSE risk of rearing insects on residual streams containing animal proteins. We used a risk assessment approach similar to EFSA's evaluations of the BSE risk of PAPs and ruminant collagen and gelatine (EFSA, 2018, 2020, 2024b), accounting for the possible pathways along which cattle could be exposed to BSE infectivity if residual streams would be contaminated with BSE. Under the assumption that feeding of PAPs, including insect meal, to ruminants remains prohibited in the EU, exposure of cattle to BSE-contaminated insect meal will only be indirect. Exposure routes considered in the risk assessment were cross-contamination of ruminant feed, grassland fertilized with animal manure derived from non-ruminants (pigs or poultry) or insects (frass), and calf milk replacer.

## 2 Material and methods

### 2.1 Residual streams and insect species

Three residual streams were evaluated for their BSE risk if used as a substrate for insect rearing: former food from supermarkets (SM), kitchen food scraps from consumer households (KF), and category 2 meat meal from animal rendering (MM). Each of these materials is currently unauthorized in the EU for applications in feed for any livestock animal, including insects. Former food from supermarkets and kitchen food scraps are considered a mix of plant-based and animal-based food that might include bovine meat products. Category 2 meat meal is a product from the rendering industry containing PAPs derived from ruminants and non-ruminants that were not slaughtered for human consumption (EC, 2009). We assumed category 2 meat meal is produced from animals declared unfit for human consumption at the slaughterhouse and does not include fallen stock, i.e., animals that died on the farm or during transport, nor animals subjected to emergency slaughter. The animals processed into category 2 meat meal are thus not subjected to testing for BSE (EC, 2001; WOAH, 2023).

Two insect species were considered: yellow mealworm (*Tenebrio molitor*) (YMW) and black soldier fly (*Hermetia illucens*) larvae (BSFL). These insect species are the most cultivated worldwide for feed, and especially BSFL are considered promising insects to valorize waste and by-products of the food production system (Athanassiou et al., 2024; Doughty et al., 2024).

### 2.2 Model outline

A quantitative, stochastic risk model was built in Excel and @Risk 8.3.2 (Lumivero, 2025) to estimate the BSE infectivity that could potentially reach cattle if each of the residual streams identified above were used as substrate for insect rearing, and the corresponding probability that this will result in at least one new BSE infection. The BSE infectivity is given as cattle oral ID_50_ (CoID_50_), where 1 CoID_50_ equals the amount of infectivity at which 50% of the exposed animals will get infected. In the model calculations, we assumed that a single BSE-infected cow at clinical end-state escaped on-farm and slaughterhouse detection and is processed either into meat products for human consumption or into category 2 meat meal if the animal is declared unfit for human consumption for other reasons than the undetected BSE infection. The meat products for human consumption will only end up in substrate for insects if discarded at retail (SM) or at the household level (KF). Exposure routes along which the infectivity in the residual streams could reach cattle are (1) cross-contamination of ruminant feed at the feed mill, during transport or at the livestock farm if insect meal is processed into feed for pigs or poultry; (2) intake of grass or forage crops that have been fertilized with frass or manure from pigs or poultry that were fed insect meal; and (3) consumption of calf milk replacer if the insect meal is used in aquafeed, after which fishmeal produced from this fish is used as an ingredient in calf milk replacer (De Vos and Heres, 2009; EFSA, 2018; De Vos et al., 2024). To assess the BSE infectivity reaching cattle, the reduction of infectivity along the production chain was evaluated using mass-balance equations. This reduction is either achieved by inactivation of prions due to processing (e.g., rendering) or by ‘partial removal' of prions from the production chain if products are used such that infectivity can no longer reach cattle. All steps in the model–from BSE-infected cow to exposure of cattle to infectivity– are given in the flowchart of Figure 1. An overview of all input parameters used in the risk model and their estimated values is given in Table 1.

**Figure 1:**
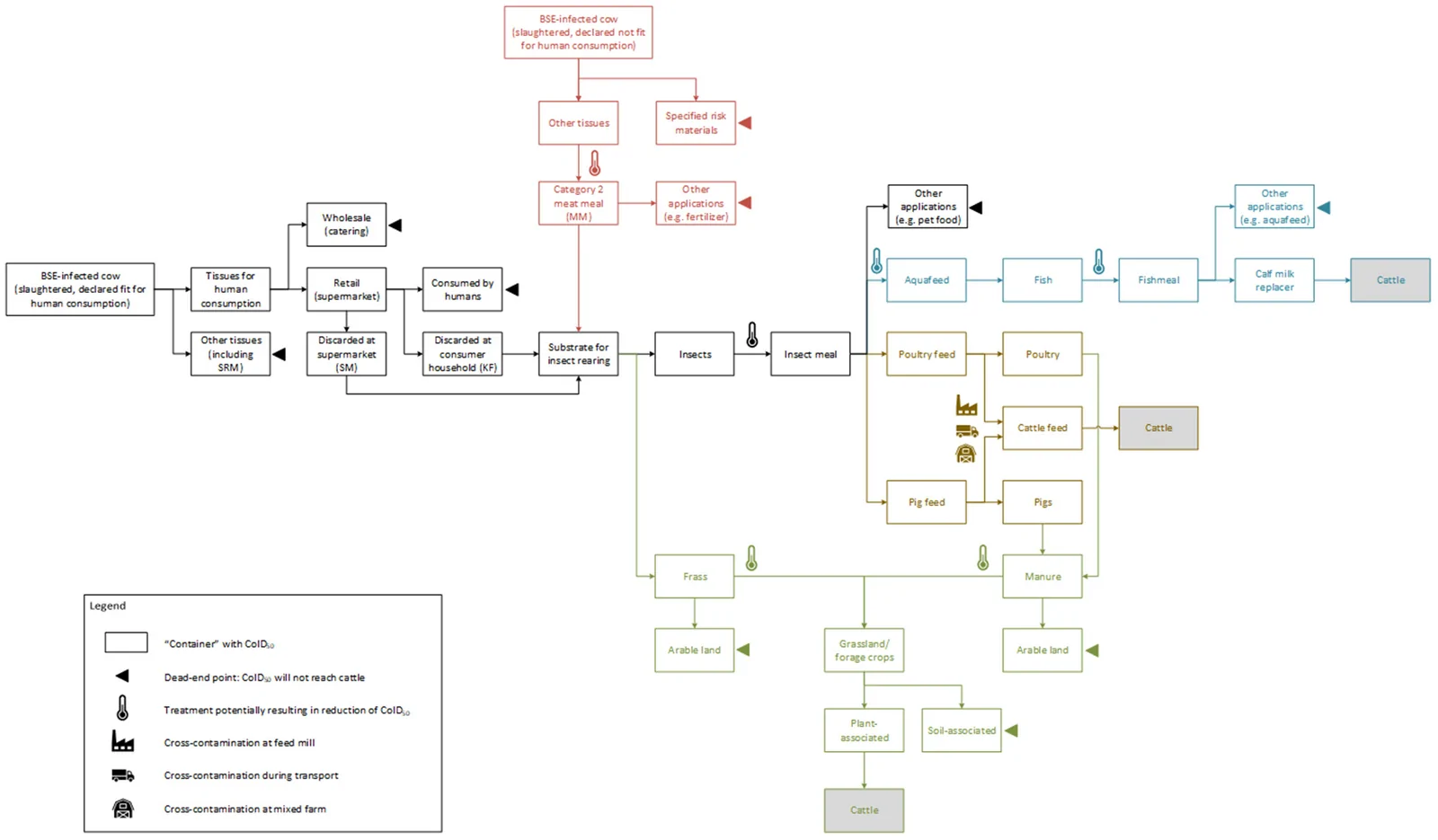
Flowchart presenting the pathways from a slaughtered BSE infected cow to exposure of cattle to BSE infectivity if former food from supermarkets (SM), kitchen food scraps from consumer households (KF) or category 2 meat meal (MM) would be used as a substrate for insect growing. Colors indicate the different sources of infectivity (black: BSE-infected cow declared fit for human consumption; red: BSE-infected cow declared not fit for human consumption) and the different exposure routes (brown: contaminated ruminant feed; green: grassland fertilized with pig or poultry manure or frass; blue: calf milk replacer). SRM=specified risk materials.

**Table 1:** Input parameters of the risk model to assess the BSE exposure of cattle if residual streams containing animal proteins are used as a substrate for insect growing.

Description of input parameter	Input parameter	Value	Unit	Source
Live weight of a slaughtered adult bovine	*W*_*bovine*_	Normal (597, 114, Truncate(0))	kg	Ballarin et al. (2016)
Weight of edible products as a proportion of total weight of a slaughtered adult bovine	*F*_*edible*_	Pert(0.5, 0.55, 0.6)	-	Cummins and Adkin (2007); Hicks and Verbeek (2016); Alao et al. (2017)
Weight of hide as a proportion of total weight of a slaughtered adult bovine	*F*_*hide*_	Uniform(0.07, 0.09)	-	Hicks and Verbeek (2016); EFSA (2020); Vion (2024); Darling Ingredients, pers. comm.
Weight of brain	*W*_*brain*_	Normal (480.5, 47.8, Truncate(0))	g	Ballarin et al. (2016)
Weight of spinal cord	*W*_*sc*_	Uniform(200, 306.9)	g	EFSA (2024b)
Weight of dorsal root ganglia (DRG)	*W*_*DRG*_	30	g	EFSA (2018)
Weight of intestines and mesenteries (I&M)	*W*_*IM*_	Pert(6290, 6612, 6807)	g	EFSA (2014a)
Weight of head	*W*_*head*_	Normal(22.48, 2.39, Truncate(0, 27.32)	kg	Anderson et al. (2025)
BSE concentration in specified risk materials (SRM)^a^	*C*_*SRM*_	LognormalAlt(2.5 %=1.25, 50 %=6.66, 97.5 %=33.3)^b^	CoID_50_/g	Konold et al. (2012b); EFSA (2020, 2024b)
BSE concentration in dorsal root ganglia (DRG)	*C*_*DRG*_	10^∧^(Log_10_(*C*_*SRM*_) – Uniform(1,1.5))	CoID_50_/g	Arnold et al. (2009); EFSA (2018)
BSE infectivity in intestines and mesenteries (I&M)	*I*_*I&M*_	Lognormal(2.8, 10.7, Truncate(0,1479)	CoID_50_	EFSA (2018)
BSE concentration in edible tissues	*C*_*edible*_	10^−6.4^	CoID_50_/g	Wells et al. (2007); Konold et al. (2012b); De Vos et al. (2024)
BSE concentration in tissues processed into category 2 meat meal (including edible tissues)	*C*_*cat*2_	10^−6.4^	CoID_50_/g	Wells et al. (2007); Konold et al. (2012b); De Vos et al. (2024)
Probability of incomplete removal of spinal cord	*P*_*sc*_	0.01	-	EFSA (2020)
Fraction of spinal cord not removed if incomplete removal	*F*_*sc*_	0.025	-	EFSA (2020)
Probability of brain tissue embolism by stunning	*P*_*emb*_	Beta(7, 198)	-	Coore et al. (2005); Wagner et al. (2019)
Weight of brain tissue embolism	*W*_*emb*_	Normal (1.34, 1.03, Truncate(0))	g	EFSA (2004)
Fraction of brain tissue embolism in edible tissue	*F*_*emb*_	0.1	-	Estimate
Reduction of BSE infectivity by rendering (133 degrees, 3 bar, 20 min)	*R*_*rend*_	10^∧^Pert(1, 2.3, 3)	-	Schreuder et al. (1998); EFSA (2018)
Fraction of category 2 meat meal used as substrate for insect growing	*F*_*cat*2_*IR*_	0.05	-	Darling Ingredients, pers. comm.
Fraction of bovine meat products to retail	*F*_*retail*_	0.54	-	Dagevos et al. (2023)
Fraction of meat products^c^ discarded at supermarket (not sold)	*F*_*disc*_*SM*_	0.014	-	STV (2024)
Fraction of meat products^c^ discarded as kitchen food scraps at household level (not consumed)	*F*_*disc*_*KF*_	0.0345	-	Van Lieshout and Knüppe (2023)
Fraction of former food from supermarkets used as substrate for insect growing	*F*_*SM*_*IR*_	0.9	-	Nijsen company, pers. comm.
Fraction of kitchen food scraps from consumer households used as substrate for insect growing	*F*_*KF*_*IR*_	0.5	-	Assumption
Weight fraction of insects at harvest (defined as weight of insects divided by total weight of insects and frass)	*F*_*insec*_*t*__*i, s*__	For *i* = BSFL and *s* = SM: Normal(0.29, 0.011) For *i* = BSFL and *s* = KF: Normal(0.19,0.019) For *i*=BSFL and *s* = MM: 0.05 × Normal(0.34,0.036)^d^ For *i* = YMW and *s* = SM: Normal(0.31,0.010) For *i* = YMW and *s* = KF: Normal(0.39, 0.0097) For *i* = YMW and *s* = MM: Normal(0.49, 0.015)	-	Den Boer and Brugmans (2022); Meijer et al. (2026); Hoek-van den Hil et al., in prep
Reduction of BSE infectivity by processing insects into insect meal	*R*_*ins*_*meal*_	1	-	Schreuder et al. (1998)
Reduction of BSE infectivity by heat treatment of frass (70^o^C for 60 min)	*R*_*frass*_	1	-	Schreuder et al. (1998)
Expected market share of insect meal for pig and poultry feed	*F*_*pp*_*feed*_	Uniform(0.074, 0.077)	-	IPIFF (2023)
Carry-over fraction for cross-contamination at feed mill, transport and farm	*F*_*cc*_	10^−3^	-	EFSA (2018)
Fraction of frass applied to grassland and forage crops	*F*_*frass*_	0.05	-	Assumption based on Blanken et al. (2022)
Fraction of pig and poultry manure applied to grassland and forage crops	*F*_*manure*_	0.2	-	Assumption
Reduction of BSE infectivity in gastro-intestinal tract of pigs and poultry	*R*_*pp*_*GI*_	1	-	Assumption based on De Vos and Heres (2009)
Annual production of grass or forage crops	*P*_*grass*_	Uniform(9000,11000)	kg	Agrimatie (2024)
Fraction of soil ingested with grass or forage crops	*F*_*soil*_	Uniform(0.01,0.0234)	-	De Vos and Heres (2009)
Density of the soil	*D*_*soil*_	Uniform(1500, 1600)	kg/m^3^	De Vos and Heres (2009)
Area on which frass or manure is applied	*Area*	10,000	m^2^	Standardized to one hectare
Depth at which frass or manure is applied	*Depth*	Pert(0.01, 0.05, 0.15)	m	De Vos and Heres (2009)
Expected market share of insect meal for aquafeed	*F*_*aquafeed*_	Uniform(0.1111, 0.3661)	-	IPIFF (2023)
Reduction of BSE infectivity by extrusion of aquafeed	*R*_*aquafeed*_	10^∧^Pert(0.1, 0.2, 1)	-	Schreuder et al. (1998); Sørensen (2012); De Vos et al. (2024)
Probability that BSE infectivity is retained in the gastro-intestinal tract of fish	*P*_*fish*_*GI*_	Beta(s+1, n-s+1)^e^	-	Estimate based on Ingrosso et al. (2006)
Time period during which infectivity can be recovered from the gastro- intestinal tract of fish	*T*_*fish*_*GI*_	Discrete Uniform(1, 15, 30, 60, 90)	day	Estimate based on Ingrosso et al. (2006)
Length of fasting period before harvesting fish	*T*_*fish*_*fast*_	$Min{\left(Pert\left(1,1,2\right),\;T_{fish\text{\_}GI}\right)}^{\text{f}}$	day	Alltech Coppens (2023)
Rearing period of fish	*T*_*fish*_*rear*_	365	day	Alltech Coppens (2023)
Fraction of farmed fish that is byproducts	*F*_*fish*_*bp*_	0.45	-	Aspevik et al. (2017)
Reduction of BSE infectivity when processing fish byproducts into fishmeal	*R*_*fm*_	1	-	FAO (1986); Schreuder et al. (1998); De Vos et al. (2024)
Fraction of produced fishmeal processed into calf milk replacer	*F*_*CMR*_	0.05	-	De Vos et al. (2024)
Dose-response parameter	α	$\frac{\ln\;2}{ID50}$ (where *ID*50 equals 1 CoID_50_)	CoID50-1	Haas et al. (1999)

^a^Based on estimates of infectivity of the central nervous system including brain and spinal cord.

^b^Lognormal distribution with median value of 6.66 and 95 % confidence interval between 1.25 and 33.3.

^c^Only data available for meat in general, not specifically for bovine meat.

^d^Relative yield of BSFL reared on category 2 meat meal if compared to BSFL reared on chicken feed (Den Boer and Brugmans, 2022) multiplied by the weight fraction of insects at harvest if reared on chicken feed (Hoek-van den Hil et al., in prep).

^e^The values of *s* (number of fish tested positive) and *n* (total number of fish tested) were dependent on the value of *T*_*fish*_*GI*_ and based on results for rainbow trout only. If *T*_*fish*_*GI*_=1: *s* = 1 and *n* = 8; If *T*_*fish*_*GI*_=15: *s* = 0; *n* = 8; If *T*_*fish*_*GI*_=30: *s* = 0, *n* = 8; If *T*_*fish*_*GI*_=60: *s* = 0, *n* = 6; If *T*_*fish*_*GI*_=90: *s* = 0, *n* = 11. Furthermore, the sampled value of *P*_*fish*_*GI*_ at *T*_*fish*_*GI*_ = *i* was set conditional on the value of *P*_*fish*_*GI*_ at *T*_*fish*_*GI*_ = *i*−1 to ensure a decreasing probability over time, i.e. *P*_*fish*_*GI*_ = *Min*(_*P*_*fish*_*GI*_*T*_*fis*_*h*__*GI*__ = *i*_,_*P*_*fish*_*GI*_*T*_*fis*_*h*__*GI*__ = *i*−1_ ).

^f^The minimum of the Pert(1, 1, 2) distribution and *T*_*fish*_*GI*_ was taken to avoid negative values if *T*_*fish*_*GI*_=1.

### 2.3 Assumptions

In the model calculations, we assumed a homogenous distribution of BSE infectivity in both the contaminated tissues of an infected animal, and the different products produced along the pathway from slaughtered cow to ingestion by cattle (Figure 1). We did not differentiate between the risk posed by cattle infected with the classical variant of BSE (C-BSE) or the atypical variants (L-BSE and H-BSE).

The risk model was built to represent the BSE risk of rearing insects on residual streams containing animal proteins in the current European situation. However, data used to quantify input parameters was sometimes limited to a single European country or derived from historical census data. We assumed this data to be representative for the geographical and temporal scope of the model.

### 2.4 Model calculations

#### 2.4.1 BSE infectivity in residual streams

The BSE infectivity in each of the residual streams depends on the infectivity in the different tissues of the slaughtered cow, the removal of specified risk materials (SRM), the probabilities of incomplete removal of SRM and cross-contamination at the slaughterhouse, and the estimated fraction of the carcass that ends up in the residual stream.

BSE infectivity in infected cattle is primarily present in SRM, which are mostly tissues of the central nervous system including the brain and the spinal cord. Removal of SRM at slaughter is an important measure to control the BSE risk. The tissues removed depend on the age of the animal and the BSE risk status of the country (EC, 2001). Since all EU member states except Greece now have a negligible risk status for BSE (EC, 2022), we assumed removal of SRM as prescribed for negligible risk countries. This includes removal of the skull including the brain and eyes and the spinal cord of bovine animals aged over 12 months. The vertebral column, the dorsal root ganglia (DRG) and the intestines and mesenteries (I&M) are, however, retained with the carcass, despite presence of low levels of infectivity in these tissues (EFSA, 2018, 2020). We assumed that all of these tissues are used for food production, whereas in reality part of it might also be used for other applications or processed as category 3 byproducts.

Low levels of BSE infectivity may also be present in other bovine tissues (EFSA, 2018). There is some evidence that BSE infectivity can be present in muscle tissue, especially in the muscle spindles of skeletal muscles, although this is less likely in asymptomatic cattle (Buschmann and Groschup, 2005; Wells et al., 2005; Konold et al., 2012a; Suardi et al., 2012; Okada et al., 2014). However, not all studies that investigated muscle tissue were able to confirm the presence of prions (PrP^Sc^) (Wells et al., 2005; Espinosa et al., 2007; Franz et al., 2012). To the best of our knowledge, no quantitative estimates of the infectivity levels in muscle tissue are available. In the baseline model calculations, we used a worst-case approach and assumed presence of BSE infectivity in edible tissues just below the detection limit of the bioassays used, which was set to 10^−6.4^ CoID_50_/g (Wells et al., 2007; Sohn et al., 2009; Konold et al., 2012b; De Vos et al., 2024).

Another source of infectivity in the products harvested for human consumption is cross-contamination at the slaughterhouse due to incomplete removal of SRM or brain tissue embolism caused by stunning when using a penetrating captive bolt gun (EFSA, 2018, 2020; De Vos et al., 2024). EFSA (2020) assumed that in 1% of the slaughtered animals approximately 2.5% of the spinal cord may remain attached to the vertebrae and enter the production process. Studies by Coore et al. (2005) and Wagner et al. (2019) indicated that the probability of brain tissue embolism at stunning is approximately 3%. This is estimated to result in 1.34±0.23 (SE) gram of brain tissue being introduced into the venous circulation (EFSA, 2004). Part of this infectivity could be traversed to the heart and lungs or other organs when blood circulation is not stopped quickly after stunning (Ramantanis et al., 2005; EFSA, 2007).

Slaughter of cattle results in edible and non-edible products, the latter being processed as by-products (with the exception of the SRM). Approximately 55% of the bovine carcass yields products for human consumption (Hicks and Verbeek, 2016; Alao et al., 2017). When a slaughtered bovine is declared unfit for human consumption, the whole carcass will be processed as category 2 meat meal except for the SRM and the hide. For the model calculations, we assumed that the entire head including the brains, eyes and other potentially infectious tissues such as the tonsils and trigeminal ganglia, will be removed from the carcass, together with the spinal cord.

The total BSE infectivity (CoID_50_) present in the edible products (meat and edible organs) of a BSE-infected cow declared fit for human consumption (*I*_*meat*_) is simulated as:

$$I_{meat}\;=\;C_{edible}\;\times \;\left(W_{bovine}\;\times \;F_{edible}\;\times \;10^{3}\;-W_{DRG}-\;W_{IM}\;\right)+\;C_{DRG}\;\times \;W_{DRG}+\;I_{I\;\&\;M}\;+\;C_{SRM}\times \;(Bernoulli\left(P_{sc}\;\right)\times W_{sc}\;\times \;F_{sc}\;+\;Bernoulli\left(P_{emb}\;\right)$$

$$\times \;W_{emb}\;\times \;F_{emb})$$ (1)

Where *C*_*edible*_, *C*_*DRG*_ and *C*_*SRM*_ is the BSE concentration (CoID_50_/g) in edible tissue, DRG and SRM (brain and spinal cord), respectively; *I*_*I&M*_ is the BSE infectivity (CoID_50_) in I&M; *W*_*bovine*_, *W*_*DRG*_, *W*_*sc*_ and *W*_*emb*_ are the weights of a bovine at slaughter (kg), the DRG (g), the spinal cord (g), and the brain tissue embolism (g), respectively; *F*_*edible*_ is the fraction of edible products harvested from a slaughtered bovine expressed as a proportion of its total weight; *P*_*sc*_ and *P*_*emb*_ are the probability of incomplete removal of the spinal cord and the probability of brain tissue embolism by stunning, respectively; and *F*_*sc*_ and *F*_*emb*_ the fraction of spinal cord and brain tissue embolism, respectively, that contaminate the edible part of the carcass. The Bernoulli distributions used to model the cross-contamination events either switch the events on or off, i.e., cross-contamination happens or it does not happen, with the frequency of cross-contamination in the model iterations being determined by the probability estimate used in the Bernoulli distribution (here: *P*_*sc*_ and *P*_*emb*_). The occurrence of both cross-contamination events was modeled independently, as they were related to different steps in the slaughtering process.

The total BSE infectivity (CoID_50_) present in the tissues of a BSE-infected cow declared unfit for human consumption and processed into category 2 meat meal (*I*_*cat*2_) is simulated as:

$$I_{cat2}=C_{cat2}\times \left(\left(W_{bovine}\times \left(1-F_{hide}\right)-W_{head}\right)\times 10^{3}-W_{sc}-W_{DRG}-W_{IM}\right)+C_{DRG}\times W_{DRG}+I_{I\&M}+C_{SRM}\times (Bernoulli\left(P_{sc}\right)\times W_{sc}\times F_{sc}+Bernoulli\left(P_{emb}\right)$$

$$\times \;W_{emb}\;\times \;F_{emb})$$ (2)

Where *C*_*cat*2_ is the BSE concentration (CoID_50_/g) in tissues processed into category 2 meat meal (including edible tissues), *F*_*hide*_ is the weight of the hide expressed as a fraction of the total weight of a slaughtered bovine, and *W*_*head*_ is the weight of the head (kg).

The outcome of Equations 1, 2 is used to estimate the infectivity in each of the residual streams. To produce category 2 meat meal, the bovine tissues harvested at slaughter are rendered, i.e., heated under pressure (133^o^C, 20 min, 3 bar; processing method 1 as indicated in Annex IV Chapter III of EC, 2011). This is expected to result in a 1 to 3 log_10_ reduction of infectivity (Schreuder et al., 1998; EFSA, 2018). Also, not all category 2 meat meal will be sold as substrate for insect rearing once this is legally permitted. The insect industry is relatively small compared to the volumes of category 2 meat meal produced and we assumed that approximately 5% of all category 2 meat meal produced can be put on the market as substrate for insect rearing. The BSE infectivity (CoID_50_) ending up in this substrate (*I*_*MM*_) is thus calculated as:

$$\begin{array}{l} I_{MM}=I_{cat2}\times {R_{rend}}^{-1}\times F_{cat2\text{\_}IR}\; \end{array}$$ (3)

Where *R*_*rend*_ is the reduction of BSE infectivity by rendering and *F*_*cat*2_*IR*_ is the fraction of category 2 meat meal that is expected to be used as a substrate for insect rearing.

The BSE infectivity in former food from supermarkets and kitchen food scraps from consumer households depends on the fraction of bovine meat products sold at retail if compared to restaurants and catering (*F*_*retail*_), the fraction of products not sold at the supermarket (*F*_*disc*_*SM*_), and the fraction of products not consumed at the household level and disposed of as ‘vegetable, fruit and food scraps' (*F*_*disc*_*KF*_). Estimates for these values were based on data from Dutch monitoring programmes on meat consumption (Dagevos et al., 2023) and food waste (Van Lieshout and Knüppe, 2023; STV, 2024). Also, not all former food from supermarkets and kitchen food scraps are suitable for use as substrate for insect rearing. We assumed that approximately 90% of former food from supermarkets can be put on the market as substrate for insect rearing once legally permitted. This share is likely to be lower for kitchen food scraps, due to, among other things, quality and logistical issues, where we assumed an inclusion rate of 50%. The BSE infectivity (CoID_50_) in substrate derived from former food from supermarkets (*I*_*SM*_) is then calculated as:

$$\begin{array}{l} I_{SM}=I_{meat}\times F_{retail}\times F_{disc\text{\_}SM}\times F_{SM\text{\_}IR}\; \end{array}$$ (4)

The BSE infectivity (CoID_50_) in substrate derived from kitchen food scraps from consumer households (*I*_*KF*_) is then calculated as:

$$\begin{array}{l} I_{KF}=I_{meat}\times F_{retail}\times \left(1-F_{disc\text{\_}SM}\right)\times F_{disc\text{\_}KF}\times F_{KF\text{\_}IR}\; \end{array}$$ (5)

#### 2.4.2 BSE infectivity in insect meal and frass

Prions can be recovered from insects after infection (Post et al., 1999; EFSA, 2015; Anonymous, 2019; Benestad et al., 2024). However, it is deemed unlikely that prions will replicate in insects, as insects lack a natural prion protein (Post et al., 1999; Bujdoso et al., 2023; Benestad et al., 2024), which is a prerequisite for replication of the infectious prions in the animal host (Marín-Moreno et al., 2017). To the best of our knowledge, there are no studies describing inactivation of prions in insects. Therefore, we assumed no inactivation of prions during insect rearing as a worst-case assumption. In the baseline model calculations we assumed equal distribution of the prions across the insects and the frass at harvest based on the relative weights of both outputs. However, the ratio of insects to frass is dependent on the substrate used to raise the insects. Relative weights of insects and frass at the end of the rearing period (21 days for YMW and 7 days for BSFL) were obtained from growth performance studies on category 2 meat meal (MM), former food from supermarkets (SM), and kitchen food scraps from consumer households (KF) (Meijer et al., 2026; Hoek-van den Hil et al., 2024) (Table 1; Supplementary Table 1). No data were available for BSFL raised on category 2 meat meal, as a pilot study indicated that this insect species showed very poor growth on category 2 meat meal with the weight of the larvae at harvest being only 5% of the weight of larvae if reared on conventional substrate (chicken feed) (Den Boer and Brugmans, 2022).

Processing of insects into insect meal mostly includes thermal processing (blanching) or drying or combinations of these two at temperatures ≤ 100 ^o^C for limited time periods, although some of the described processing methods are more rigorous (Melgar-Lalanne et al., 2019; Liceaga, 2021; Parniakov et al., 2021). The conditions of most processes are, however, not expected to result in any reduction of BSE infectivity (Schreuder et al., 1998). Therefore, in the baseline model calculations we assumed no decrease of BSE infectivity by insect meal processing.

The infectivity in insect meal _*I*_*ins*_*meal*_*i, s*_ for each insect species *i* and substrate *s* is then calculated as:

$$\begin{array}{l} {I_{ins\text{\_}meal}}_{i,s}=I_{s}\times {F_{insect}}_{i,s}\times {R_{ins\text{\_}meal}}^{-1}\; \end{array}$$ (6)

Where, *I*_*s*_ is the infectivity in each of the substrates MM, SM and KF as calculated in Equations 3-5, _*F*_*insect*_*i, s*_ is the weight fraction of insect species *i* at harvest (expressed as fraction of the total weight of insects and frass) if raised on substrate *s*, and *R*_*ins*_*meal*_ is the reduction of BSE infectivity when processing insects into insect meal (set to 1 as we assumed no reduction at all).

European regulations require heat treatment of frass at a minimum of 70 ^o^C for at least 60 min before trade (Regulation 2021/1925 amending Regulation (EU) 142/2011; EC, 2011, 2021a). The BSE risk of treated frass is estimated to be equal to untreated frass, as the prescribed heat treatment will not result in any reduction of BSE infectivity (Schreuder et al., 1998).

The infectivity in frass _*I*_*frass*_*i, s*_ for each insect species *i* and substrate *s* is then calculated as:

$$\begin{array}{l} {I_{frass}}_{i,s}=I_{s}\times \left(1-{F_{insect}}_{i,s}\right)\times {R_{frass}}^{-1}\; \end{array}$$ (7)

Where, *R*_*frass*_ is the reduction of BSE infectivity due to heat treatment of frass before trade (set to 1 as we assumed no reduction at all).

#### 2.4.3 Exposure of cattle to BSE infectivity via ruminant feed

Insect meal is not allowed for inclusion in ruminant feed. However, since 2021 it can be used as an ingredient in pig and poultry feed (Regulation (EU) 2021/1372 amending Regulation (EC) No 999/2001; EC, 2001, 2021b). This has opened up new markets for the sale of insect meal, and the expected market share of pig and poultry feed was estimated at 7.4% and 7.7%, respectively, for the years 2025 and 2030 (IPIFF, 2023).

Insect meal used in pig and poultry feed can reach cattle via cross-contamination at the feed mill, during transport, or at the livestock farm. The probabilities of cross-contamination at the feed mill are considered very low. According to Regulation (EU) 2021/1372, compound feed containing processed animal proteins derived from farmed insects is to be produced in feed mills dedicated to the production of feed for aquaculture animals, poultry or porcine animals (EC, 2021b). Production of ruminant feed at the same location is only allowed if manufacturing, storage, transport and packaging is physically separated from the production of compound feed for non-ruminants. Assuming that a small proportion (< 10%) of ruminant feed is produced at the same location as non-ruminant feed, and that at some of these locations (< 10%) production processes are not strictly separated, a small proportion (< 1%) of all ruminant feed produced could potentially be cross-contaminated with non-ruminant feed. The level of cross-contamination would be highest if the same production lines would be used for the production of ruminant and non-ruminant feed without any cleaning in-between batches. Yamamoto et al. (2006) estimated the carry-over fraction in case of no cleaning at approximately 10%, and at 2% if a dry run would be performed in-between batches. This would imply a maximum level of cross-contamination between 2 × 10^−4^ and 10^−3^.

Cross-contamination during transport could occur if transport means are used for both ruminant and non-ruminant feed. EFSA (2018) considered it a reasonable assumption that some material could remain in small amounts after routine cleaning and disinfection, although thorough cleaning and disinfection will substantially reduce the probability of cross-contamination. However, no data were available to substantiate a quantitative estimate for the overall level of cross-contamination during transport.

Cross-contamination at the livestock farm is only possible at mixed farms, i.e., farms where both ruminants and non-ruminants are present. The probability of cross-contamination at the farm depends on farm management, feed storage and animal facilities, and will vary across farms and regions. To estimate the probability of non-ruminant feed delivered to mixed livestock farms, we estimated the percentage of pigs and poultry on farms in the EU where ruminants might also be present; in 2020, this was approximately 14% and 9%, respectively (Eurostat, 2024). De Vos and Heres (2009) estimated the overall carry-over fraction at farm level at 10^−4^, resulting in an overall level of cross-contamination of 10^−5^.

In their updated opinion on the BSE risk posed by processed animal proteins, EFSA used a most likely value of 1 × 10^−3^ for cross-contamination via all of these three steps in the production chain (i.e., feed mill, transport and livestock farm), and used a value of 2 × 10^−4^ for as a best-case scenario and a value of 0.02 as a worst-case scenario (EFSA, 2018). Based on the considerations given above, we have adopted the EFSA estimate of 1 × 10^−3^ for the overall probability of cross-contamination of ruminant feed with non-ruminant feed in the baseline model calculations.

The exposure of cattle to BSE infectivity via cross-contamination of ruminant feed (_*I*_*rum*_*feed*_*i, s*_) was then calculated as:

$$\begin{array}{l} {I_{rum\text{\_}feed}}_{i,s}={I_{ins\text{\_}meal}}_{i,s}\times F_{pp\text{\_}feed}\times F_{cc}\; \end{array}$$ (8)

Where _*I*_*ins*_*meal*_*i, s*_ is the infectivity in insect meal (Equation 6), *F*_*pp*_*feed*_ is the market share of insect meal for pig and poultry feed, and *F*_*cc*_ is the combined carry-over fraction of infectivity to ruminant feed due to cross-contamination at the feed mill, during transport and at the mixed farm.

#### 2.4.4 Exposure of cattle to BSE infectivity via grassland and forage crops

BSE infectivity can also reach cattle via intake of grass or forage crops that have been fertilized with frass or manure from pigs or poultry that were fed insect meal. Considering the resistance of prions to environmental influences such as temperature and pH, we assumed no inactivation of prions in the gastro-intestinal tract of pigs and poultry, which is clearly a worst-case assumption (De Vos and Heres, 2009). There is no data on the application of frass on grassland and forage crops. Although frass has a high nutritional value as a fertilizer (Houben et al., 2020; Abd Manan et al., 2024), the costs of heat treatment hamper its widespread use as a fertilizer (Blanken et al., 2022). In the baseline model calculations, we assumed a maximum of 5% of frass applied on grassland and forage crops. The application of pig and poultry manure as a fertilizer to grassland and forage crops is highly variable across countries and regions. It not only depends on the local agricultural systems, but also on regulatory frameworks setting maximum levels for land application of nutrients including nitrogen and phosphorous (Köninger et al., 2021; Ball et al., 2024). In the baseline model calculations, we assumed that on average 20% of pig and poultry manure is applied to grassland and forage crops.

The exposure of cattle via grazing was modeled using the approach given by De Vos and Heres (2009), where frass and manure, and thus the prions, are assumed to be homogenously distributed in the upper layer of the soil and intake of soil is assumed to be proportionate to the consumption of grass or forage crops. The exposure of cattle to BSE infectivity via frass (_*I*_*grass*_*frass*_*i, s*_) is thus modeled as:

$$\begin{array}{l} {I_{grass\text{\_}frass}}_{i,s}={I_{frass}}_{i,s}\times F_{frass}\times \frac{P_{grass}\times F_{soil}}{Area\times Depth\times D_{soil}}\; \end{array}$$ (9)

Where _*I*_*frass*_*i, s*_ is the infectivity in frass (Equation 7), *F*_*frass*_ is the fraction of frass applied to grassland and forage crops, *P*_*grass*_ is the annual production of grass or forage crops (kg dry matter per hectare), *F*_*soil*_ is the fraction of soil ingested with grass or forage crops, *Area* is the surface area considered (here: one hectare), *Depth* is the depth at which manure is applied, and *D*_*soil*_ is the density of the soil. The values of each of these input parameters are given in Table 1. Implicit assumptions in these calculations were (1) no inactivation of infectivity in the soil, (2) no leaching of infectivity to soil water, (3) no assimilation of infectivity by crops, and (4) consumption of all infectivity associated with grass in the first year after application of manure (i.e., no crop rotation) (De Vos and Heres, 2009).

Similarly, the exposure of cattle to BSE infectivity via pig and poultry manure (_*I*_*grass*_*manure*_*i, s*_) is modeled as:

$$\begin{array}{l} {I_{grass\text{\_}manure}}_{i,s}={I_{ins\text{\_}meal}}_{i,s}\times F_{pp\text{\_}feed}\times \left(1-F_{cc}\right)\times {R_{pp\text{\_}GI}}^{-1} \end{array}$$

$$\begin{array}{l} \times F_{manure}\times \frac{P_{grass}\times F_{soil}}{Area\times Depth\times D_{soil}}\; \end{array}$$ (10)

Where, *R*_*pp*_*GI*_ is the reduction of BSE infectivity due to inactivation of prions in the gastro-intestinal tract of pigs and poultry (set to 1 as we assumed no reduction at all) and *F*_*manure*_ is the fraction of pig and poultry manure applied to grassland and forage crops.

#### 2.4.5 Exposure of cattle to BSE infectivity via calf milk replacer

A more direct exposure route of cattle to BSE infectivity is via calf milk replacers if these would contain fishmeal derived from fish fed with insect meal. The exposure of cattle via this route was modeled using the approach given De Vos et al. (2024).

The first step was to estimate the BSE infectivity entering aquafeed. The use of insect meal as an ingredient of aquafeed in the EU was authorized in 2017. The aquafeed market has now become an important market for insect feed, with a projected market share of 11.11% and 36.61% for the years 2025 and 2030 (IPIFF, 2023). We assumed production of aquafeed by extrusion at a minimum of temperature of 120^o^C and under high pressure, which is expected to result in a slight reduction of BSE infectivity by 0.1 to 1 log_10_ (Schreuder et al., 1998). The BSE infectivity in aquafeed (_*I*_*aquafeed*_*i, s*_) is thus calculated as:

$$\begin{array}{l} {I_{aquafeed}}_{i,s}={I_{ins\text{\_}meal}}_{i,s}\times F_{aquafeed}\times {R_{aquafeed}}^{-1}\; \end{array}$$ (11)

Where, *F*_*aquafeed*_ is the market share of insect meal for aquafeed and ${R_{aquafeed}}^{-1}$ is the reduction of BSE infectivity achieved by extrusion.

The next step was to estimate the BSE infectivity present in fishmeal. Results from an experiment by Ingrosso et al. (2006) in rainbow trout (*Oncorhynchus mykiss*) and turbot (*Scophthalmus maximus*) indicate that TSE infectivity is probably quickly cleared from fish tissue. Ingrosso et al. (2006) tested several tissues for remaining infectivity at 1, 15, 30, 60 and 90 days after forced feeding of scrapie. Only in 1 out of 8 challenged rainbow trouts, infectivity could be recovered from the intestines 1 day after infection; no infectivity was recovered at later time points. These observations were used to model a time-dependent probability that BSE infectivity is retained in the gastro-intestinal tract of fish. Furthermore, a one- to two-day fasting period before harvesting was assumed (Alltech Coppens, 2023), which contributes to a further reduction of the amount of BSE infectivity in the gastro-intestinal tract of fish. Only the by-products of farmed fish are processed into fishmeal. An average of 45% by-products was assumed based on figures for global fish production (Aspevik et al., 2017). The processing conditions of fishmeal production (cooking and drying at temperature ≤ 100^o^C) (FAO, 1986) were not expected to result in a reduction of BSE infectivity (Schreuder et al., 1998). The BSE infectivity in fishmeal (_*I*_*fishmeal*_*i, s*_) was then calculated as:

$$\begin{array}{l} {I_{fishmeal}}_{i,s}={I_{aquafeed}}_{i,s}\times P_{fish\text{\_}GI}\times \frac{\left(T_{fish\text{\_}GI}-T_{fish\text{\_}fast}\right)}{T_{fish\text{\_}rear}} \end{array}$$

$$\begin{array}{l} \times F_{fish\text{\_}bp}\times {R_{fm}}^{-1}\; \end{array}$$ (12)

Where _*I*_*aquafeed*_*i, s*_ is the infectivity in aquafeed (Equation 11), *P*_*fish*_*GI*_ is the probability that BSE infectivity is retained in the gastro-intestinal tract of fish, *T*_*fish*_*GI*_ is the time period during which infectivity can be recovered from the gastro-intestinal tract, *T*_*fish*_*fast*_ is the length of the fasting period before harvesting, *T*_*fish*_*rear*_ is the rearing period of fish, *F*_*fish*_*bp*_ is the fraction of farmed fish that is considered by-products (not used for human consumption), and *R*_*fm*_ is the reduction of BSE infectivity achieved by processing fish by-products into fishmeal (set to 1 as we assumed no reduction at all).

The last step was to estimate the BSE infectivity present in calf milk replacer, which is dependent on the inclusion rate of fishmeal in calf milk replacer. The use of fishmeal in calf milk replacer is, however, limited due to economic and quality constraints. De Vos et al. (2024) assumed that only 5% of fishmeal production could be processed in calf milk replacer. The BSE infectivity in calf milk replacer (_*I*_*CMR*_*i, s*_) was then calculated as:

$$\begin{array}{l} {I_{CMR}}_{i,s}={I_{fishmeal}}_{i,s}\times F_{CMR}\; \end{array}$$ (13)

Where _*I*_*fishmeal*_*i, s*_ is the infectivity in fishmeal (Equation 12), *F*_*CMR*_ is the estimated fraction of produced fishmeal that is used as ingredient in calf milk replacer.

#### 2.4.6 Probability of new BSE infections

The probability that rearing insect species *i* on substrate *s* will result in one or more new BSE infections via exposure route *r* (_*P*_*BSE*_*r, i, s*_) was estimated using an exponential distribution, assuming that there is no threshold dose for prions to cause infection (Gale, 1998):

$$\begin{array}{l} {P_{BSE}}_{r,i,s}=1-\exp^{-\left(\alpha \times I_{r,i,s}\right)}\; \end{array}$$ (14)

Where, *I*_*r, i, s*_ is the estimated dose of BSE infectivity (CoID_50_) ingested by cattle via exposure route *r* when insect species *i* is reared on substrate *s* and α is the dose-response parameter. Note that for the exposure via cross-contamination of ruminant feed, *I*_*r, i, s*_ equals _*I*_*rum*_*feed*_*i, s*_ (Equation 8), for the exposure via grass or forage crops fertilized with contaminated frass *I*_*r, i, s*_ equals _*I*_*grass*_*frass*_*i, s*_ (Equation 9), for the exposure via grass or forage crops fertilized with contaminated pig or poultry manure *I*_*r, i, s*_ equals _*I*_*grass*_*manure*_*i, s*_ (Equation 10), and for the exposure via contaminated calf milk replacer *I*_*r, i, s*_ equals _*I*_*CMR*_*i, s*_ (Equation 13). To estimate the overall probability of at least one new BSE infection if insects species *i* is reared on substrate *s* (_*P*_*BSE*_*i, s*_), the dose in Equation 14 was estimated as the total infectivity reaching cattle via all of the individual exposure routes ($I_{i,s}=\;\underset{r}{\sum }I_{r,i,s}$).

### 2.5 Model output

Main output parameters of the model are the amounts of BSE infectivity (CoID_50_) reaching cattle when insect species *i* is reared on substrate *s*, per exposure route (*I*_*r, i, s*_) and overall (*I*_*i, s*_); and the associated probabilities of at least one new BSE infection (_*P*_*BSE*_*r, i, s*_ and _*P*_*BSE*_*i, s*_). All baseline and what-if scenarios were run using Latin Hypercube sampling. We used the Mersenne Twister random number generator with a fixed initial seed set to 1 for each simulation. Model results for each scenario were based on 10,000 iterations to account for uncertainty in model input parameters. A convergency check comparing results of multiple simulations starting with different seed values indicated that 10,000 iterations sufficed to have stable results for the scenarios with SM and KF as substrate with < 2% change in the median values and < 5% change in the 97.5 percentile values. Model convergence for the scenarios with MM as substrate was slightly lower with < 5 % change for median values and < 10% change for 97.5 percentile values due to high uncertainty on the inactivation of prions by rendering. These deviations were, however, still considered acceptable as they did not result in a change of model results on a log_10_ scale and thus did not affect the conclusions of the study.

### 2.6 Uncertainty analysis

To evaluate the impact of uncertain input parameters on model output, correlation coefficients between these input parameters and the main output parameters were calculated in @Risk. This sensitivity analysis could only be performed for uncertain input parameters that were modeled by a probability distribution. To explore the impact of other input parameters and assumptions made, several what-if scenarios were run with the model. An overview of all what-if scenarios is given in Table 2.

**Table 2:** Overview of what-if scenarios explored with the risk model.

Scenario	Assumptions challenged	Description^a^	Parameters changed	Rationale
WI-1	Infectivity in bovine carcass	Infectivity in edible tissue and tissues processed into category 2 meat meal 6 log lower than in SRM	*C*_*edible*_=10^∧^(log_10_(*C*_*SRM*_)-6); *C*_*cat*2_=10^∧^(log_10_(*C*_*SRM*_)-6)	Buschmann and Groschup, 2005
WI-2	Infectivity in bovine carcass	Infectivity in edible tissue and tissues processed into category 2 meat meal set to zero	*C*_*edible*_=0; *C*_*cat*2_=0	Infectivity only found in muscle spindles; these are considered part of peripheral nervous system
WI-3	Infectivity in bovine carcass	Infectivity in DRG as given by (EFSA 2020, 2024b)	*C*_*DRG*_ = *C*_*SRM*_	EFSA, 2020, 2024b
WI-4	Infectivity in bovine carcass	Removal of DRG and I&M as in countries with controlled risk status	*W*_*DRG*_ = *Bernoulli*(*P*_*sc*_) × *W*_*DRG*_×*F*_*sc*_; *I*_*I&M*_ = 0	EC, 2001; EFSA, 2020
WI-5	Infectivity in bovine carcass	No removal of SRM at all	*P*_*sc*_=1; *F*_*sc*_=1; *P*_*emb*_=1; *F*_*emb*_=1; *W*_*emb*_ = *W*_*brain*_	Worst-case scenario
WI-6	Infectivity in insect meal	All infectivity retained in insects	*F*_*insec*_*t*__*i, s*__=1	Assumption
WI-7	Infectivity in insect meal	All infectivity accumulated in frass	*F*_*insec*_*t*__*i, s*__=0	Assumption
WI-8	Infectivity in insect meal	Only infectivity in gut contents of insects	*F*_*insec*_*t*__*i, s*__ = 0.25 × *F*_*insec*_*t*__*i, s*__	Spranghers, 2017; Tegtmeier et al., 2021
WI-9	Infectivity in insect meal	Slight inactivation by insect meal processing	*R*_*ins*_*meal*_=10^∧^Pert(0.1,0.2,1)	Inactivation assumed equal to extrusion of aquafeed
WI-10	Exposure via feed	Low level of cross-contamination of feed	*F*_*cc*_=2 × 10^−4^	EFSA, 2018
WI-11	Exposure via feed	High level of cross-contamination of feed	*F*_*cc*_=0.02	EFSA, 2018
WI-12	Exposure via feed	100% cross-contamination of feed	*F*_*cc*_=1	Worst-case scenario
WI-13	Exposure via grazing	Larger fraction of frass applied to grassland and forage crops	*F*_*frass*_=0.25	Assumption
WI-14	Exposure via grazing	Larger fraction of pig and poultry manure applied to grassland and forage crops	*F*_*manure*_=0.5	Assumption
WI-15	Exposure via grazing	Annual yield of forage crops based on corn	*P*_*grass*_=Uniform(16000,19000)	Agrimatie, 2024
WI-16	Exposure via grazing	Partial inactivation of BSE infectivity in gastrointestinal tract of pigs and poultry	*R*_*pp*_*GI*_=2	Assumption based on De Vos and Heres, 2009
WI-17	Exposure via feed and grazing	All insect meal in pig and poultry feed; all frass and pig and poultry manure applied to grassland and forage crops	*F*_*pp*_*feed*_=1; *F*_*manure*_=1; *F*_*frass*_=1	Worst-case scenario
WI-18	Exposure via calf milk replacer	All BSE infectivity retained in gastrointestinal tract of fish	*P*_*fish*_*GI*_=1; $\frac{\left(T_{fish\text{\_}GI}-T_{fish\text{\_}fast}\right)}{T_{fish\text{\_}rear}}=$1	Worst-case scenario based on De Vos et al., 2024
WI-19	Exposure via calf milk replacer	Higher reduction of BSE infectivity by extrusion of aquafeed	*R*_*aquafeed*_= 10^∧^RiskPert(2.1,2.2,2.5)	Extrusion as effective as method C of Schreuder et al., 1998
WI-20	Exposure via calf milk replacer	Larger fraction of fishmeal in calf milk replacer	*F*_*CMR*_=0.25	De Vos et al., 2024
WI-21	Exposure via calf milk replacer	All insect meal in aquafeed; all fishmeal in calf milk replacer	*F*_*aquafeed*_=1; *F*_*CMR*_=1	Worst-case scenario

^*a*^SRM, specified risk materials; DRG, dorsal root ganglia; I&M, intestines and mesenteries.

The uncertainty on BSE infectivity in bovine tissues was challenged in five what-if scenarios. In scenario WI-1, we assumed BSE infectivity in edible tissue (*C*_*edible*_) and tissues processed into category 2 meat meal (*C*_*cat*2_) to be 6 log_10_ lower than infectivity in SRM (Buschmann and Groschup, 2005), whereas in WI-2 we assumed no BSE infectivity in any other tissues of the bovine carcass than the brain, spinal cord, DRG and I&M as there is limited evidence that low levels of infectivity might be present in other tissues, including the muscle spindles of skeletal muscle (Buschmann and Groschup, 2005; Wells et al., 2005; Suardi et al., 2012; EFSA, 2014b, 2018). In scenario WI-3, we assumed infectivity levels in the DRG to equal infectivity levels in SRM. This worst-case assumption was used by EFSA in their risk assessments for ruminant collagen and gelatine (EFSA, 2020, 2024b). In scenario WI-4, we assumed removal of the vertebral column and the attached DRG, as well as the I&M as is done in countries with a controlled risk status (EC, 2001; EFSA, 2020), resulting in a lower level of infectivity in the carcass after slaughter. In this scenario, we accounted for incomplete removal of the DRG similar to the spinal cord (EFSA, 2020). In scenario WI-5, we assumed no removal of SRM at all as a worst-case scenario.

The uncertainty on BSE infectivity in insect meal was challenged in another four what-if scenarios. In scenario WI-6, we assumed all BSE infectivity present in the insects at harvest, whereas in scenario WI-7 we assumed all BSE infectivity present in the frass. In scenario WI-8, we assumed that BSE infectivity would only be present in the guts of the insects. The gut weight of insects was estimated to be approximately 25% of their body weight (Spranghers, 2017; Tegtmeier et al., 2021). In scenario WI-9, we assumed slight inactivation of BSE infectivity during processing of insect meal, similar to extrusion of aquafeed.

The uncertainty on exposure via cross-contamination of feed was challenged in three scenarios. In scenario WI-10 and WI-11, we assumed a lower and higher level of cross-contamination, respectively based on EFSA (2018). In scenario WI-12, we assumed a worst-case scenario in which all pig or poultry feed would end up with cattle (‘100% cross-contamination') due to, e.g., fraud, labeling errors or accidental access of cattle to non-ruminant feed on mixed livestock farms.

The uncertainty on exposure via grazing was challenged in four scenarios. In WI-13, we assumed a higher fraction of frass applied to grass and forage crops, whereas in WI-14 we assumed a higher fraction of pig and poultry manure applied to grass and forage crops. In WI-15, the annual yield of forage crops was based on corn rather than grass. Since the average yield of corn is higher than grass, this will result in a slightly higher intake of BSE-contaminated soil by cattle. In WI-16, we assumed partial inactivation of BSE infectivity in the gastro-intestinal tract of pigs similar to De Vos and Heres (2009).

In scenario WI-17, we challenged both our uncertainty on exposure via cross-contamination of feed and grazing by assuming a worst-case scenario in which all insect meal is used in pig and poultry feed, and all frass and pig and poultry manure is applied to grass and forage crops.

The uncertainty on exposure via calf milk replacer was challenged in the last four scenarios. We only included those what-if scenarios that had considerable impact in a previous study by De Vos et al. (2024) that evaluated the BSE risk of calf milk replacer if bovine spray-dried red blood cells would be allowed as an ingredient of aquafeed. In scenario WI-18, we assumed that all BSE infectivity would be retained in the gastro-intestinal tract of fish. This is an unlikely worst-case scenario which is not supported by the limited evidence available from experimental studies in fish (Ingrosso et al., 2006; Salta et al., 2009). In scenario WI-19, we assumed a higher reduction of infectivity by extrusion, where we assumed that inactivation by extrusion would be as efficient as heat treatment at 125 ^o^C for 15 min (method C given by Schreuder et al., 1998). In scenario WI-20, we assumed a higher fraction of fishmeal being used as an ingredient in calf milk replacer. In scenario WI-21, we assumed a worst-case scenario in which all insect meal is used in aquafeed, and all fishmeal is used as an ingredient in calf milk replacer.

## 3 Results

The BSE infectivity to which cattle are exposed depends both on the substrate and the insect species. The median infectivity ending up in each of the substrates is less than 1 CoID_50_, with the highest infectivity estimated for kitchen food scraps from consumer households and the lowest infectivity estimated for category 2 meat meal (Figure 2; Supplementary Table 2). The median overall exposure of cattle to BSE infectivity (i.e., via all exposure routes) varies from 4.7 × 10^−8^ CoID_50_ (BSFL on MM) to 5.2 × 10^−6^ CoID_50_ (YMW on KF) (Figure 3A; Supplementary Table 2). Uncertainty in results is highest for insects reared on category 2 meat meal. However, the 97.5 percentile values of exposure do not exceed 10^−4^ CoID_50_ for any of the substrate-insect combinations. The probability that insect rearing on any of these substrates results in new BSE infections is similarly low with median values varying from 3.2 × 10^−8^ (BSFL on MM) to 3.6 × 10^−6^ (YMW on KF) (Figure 3B; Supplementary Table 2). An estimated 1.1 × 10^−3^ (BSFL on MM) to 4.8 × 10^−3^ (YMW on MM) percent of the infectivity in substrate reaches cattle (Figure 4). The exposure via contaminated ruminant feed contributes most to the risk; its relative contribution is between 61% and 81% for all substrate-insect combinations but BSFL on MM (Figure 4; Supplementary Figure 1). For the latter combination, exposure to infectivity in frass via grazing has the largest contribution with 87%. This is due to the poor growth of BSFL on category 2 meat meal, resulting in a high relative weight of frass compared to insects at harvest, and therefore an assumed relatively high amount of BSE infectivity in frass. For all other substrate-insect combinations, the relative contribution of frass via grazing is only between 11% and 33%. The relative contribution of pig and poultry manure via grazing and calf milk replacer to the BSE risk is small for all substrate-insect combinations, with 0.5-3% and 1-5%, respectively.

**Figure 2:**
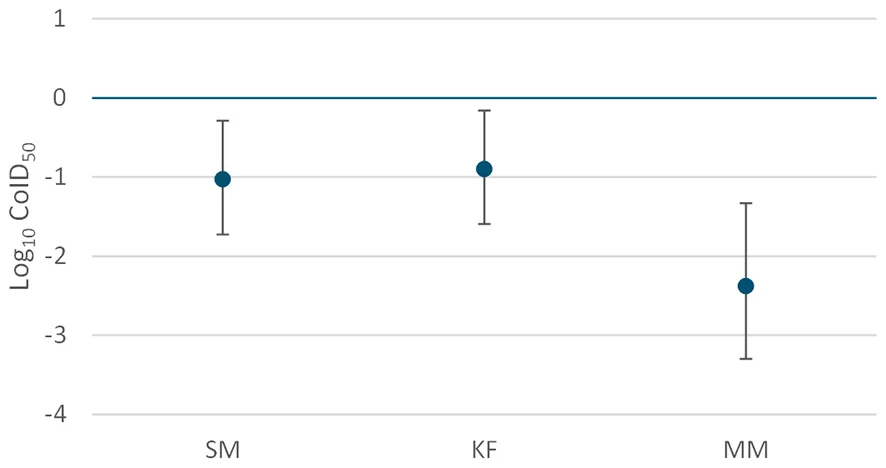
Median values and 95% uncertainty interval of the estimated BSE infectivity in substrate (log_10_ CoID_50_). SM: former food from supermarkets; KF: kitchen food scraps from consumer households; MM: category 2 meat meal.

**Figure 3:**
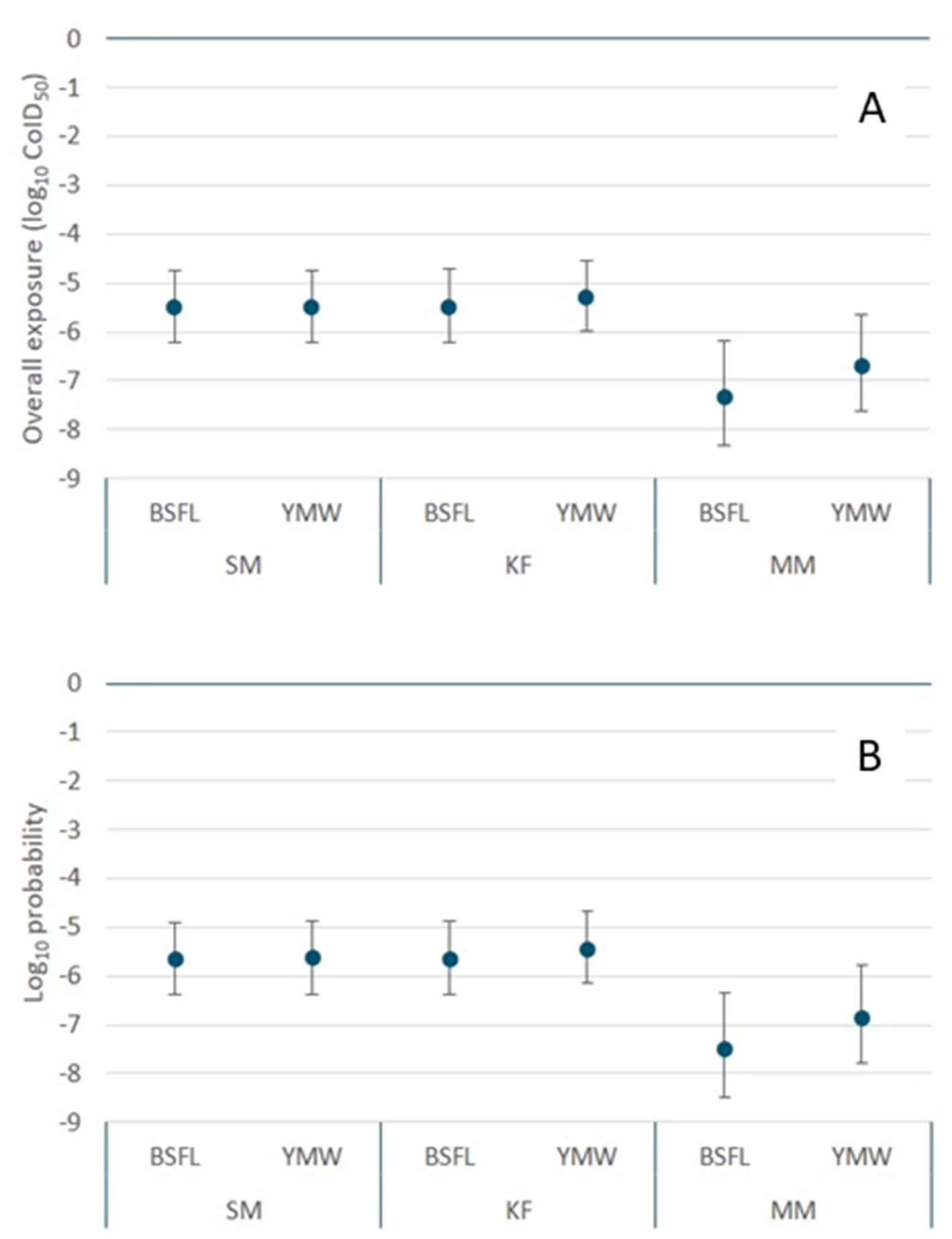
Median values and 95% uncertainty interval of the estimated BSE risk of the different substrate-insect combinations. **A)** Estimated overall exposure (log_10_ CoID_50_) of cattle to BSE infectivity. **B)** Estimated probability (log_10_ value) of ≥ 1 new BSE infection. SM: former food from supermarkets; KF: kitchen food scraps from consumer households; MM: category 2 meat meal; BSFL: black soldier fly larvae; YMW: yellow mealworm.

**Figure 4:**
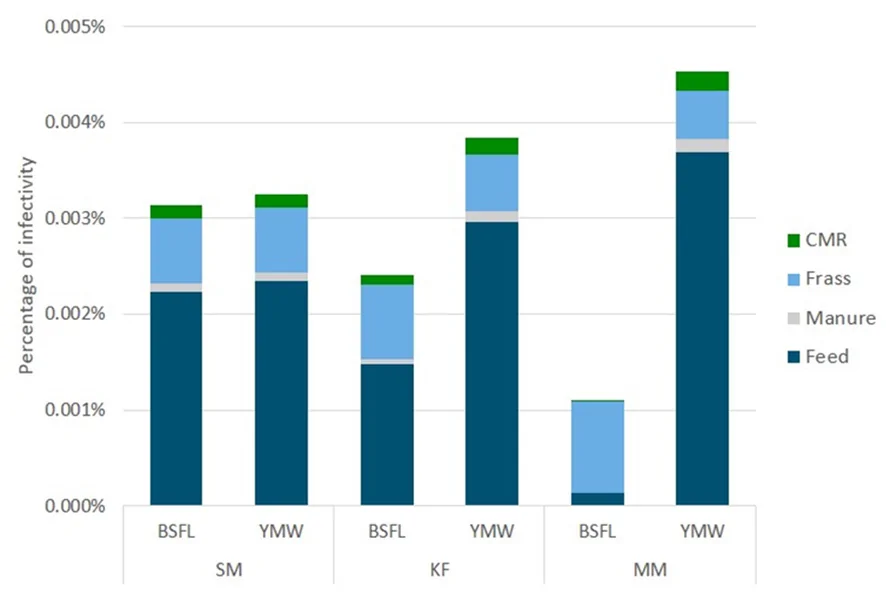
Percentage of BSE infectivity reaching cattle via the different exposure routes for each of the substrate-insect combinations. Feed: contaminated ruminant feed; Manure: grassland fertilized with pig or poultry manure via grass or forage crops; Frass: grassland fertilized with frass; CMR: calf milk replacer. SM: former food from supermarkets; KF: kitchen food scraps from consumer households; MM: category 2 meat meal; BSFL: black soldier fly larvae; YMW: yellow mealworm.

The sensitivity analysis resulted in a few uncertain input parameters that had a high correlation with the estimated overall exposure of cattle to BSE infectivity (*I*_*r, s*_) (Figure 5). Model results were most sensitive to uncertainty on input parameters estimating the BSE infectivity in edible products (*I*_*meat*_) and category 2 meat meal (*I*_*cat*2_), especially the concentration of BSE infectivity in specified risk materials (*C*_*SRM*_) and the dorsal root ganglia (*C*_*DRG*_), and the infectivity in intestines and mesenteries (*I*_*I&M*_). It is important to note here that the modeled infectivity in the DRG was dependent on the BSE infectivity in SRM (see Table 1); this explains the high correlation coefficient of *C*_*SRM*_ with the estimated overall exposure, even though cross-contamination of the carcass with brain tissue or spinal cord only occurred in a minority of the model iterations. The presence or absence of incomplete removal of the spinal cord (modeled by *Bernoulli*(*P*_*sc*_)) affected model results for all substrates but MM. Model results for MM as a substrate were, on the other hand, highly sensitive to the reduction of BSE infectivity by rendering (*R*_*rend*_). This parameter did not affect the results for the other substrates as no rendering is applied. Results for all substrate-insect combinations except for YMW on MM were sensitive to the depth at which frass or manure is applied (*Depth*). Results for BSFL on MM were also sensitive to the fraction of soil ingested with grass or forage crops (*F*_*soil*_). This is again explained by the poor growth of BSFL on MM, resulting in a high relative weight of frass compared to insects at harvest, and therefore an assumed relatively high amount of BSE infectivity in frass. This resulted in a relatively high contribution of frass via grazing to the overall risk for this substrate-insect combination (Figure 4; Supplementary Figure 1).

**Figure 5:**
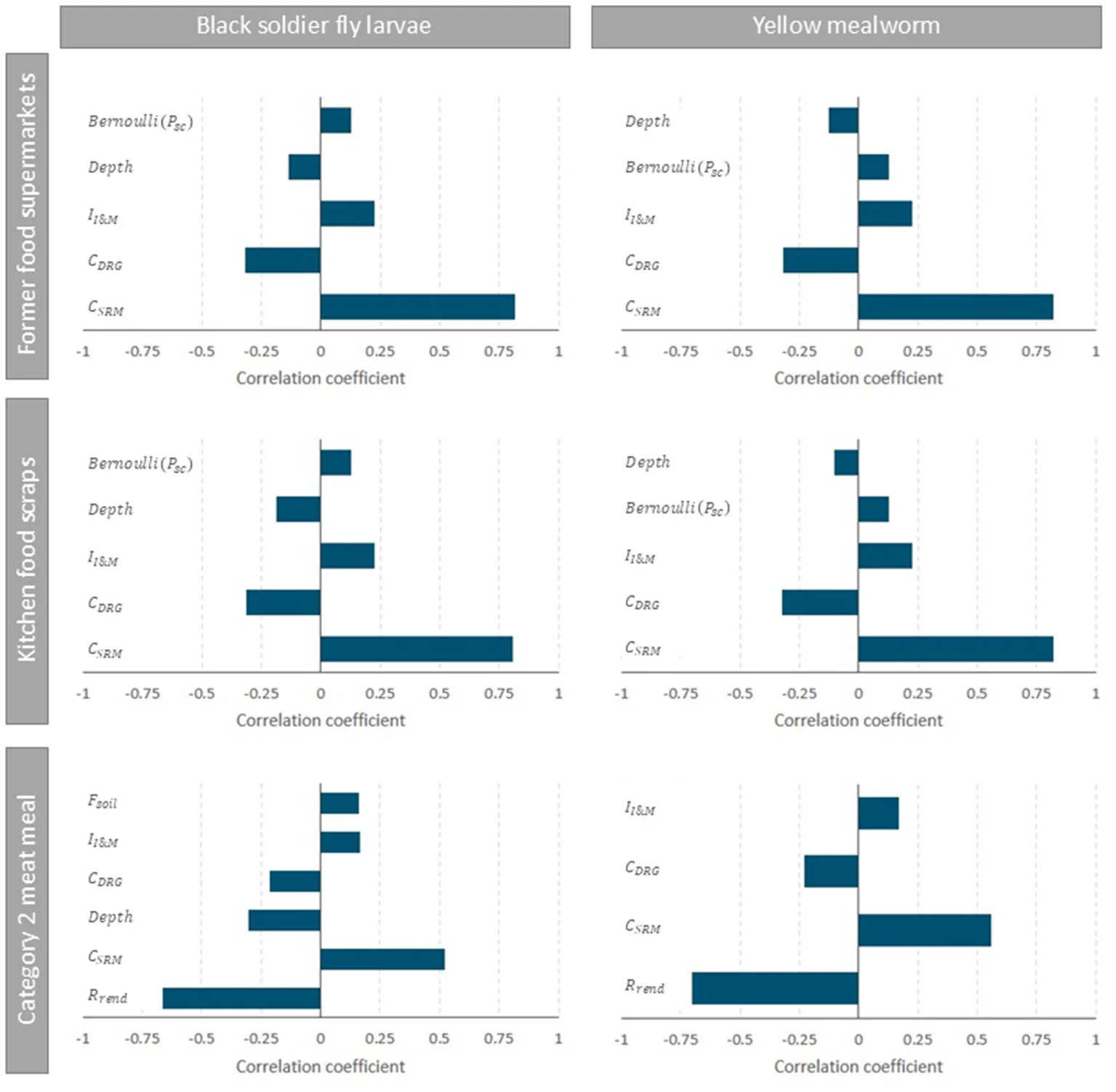
Correlation coefficients of uncertain input parameters with the estimated overall exposure of cattle to BSE infectivity. Only input parameters with a correlation coefficient > |0.1| are shown. *C*_*SRM*_ = BSE concentration in specified risk materials; *C*_*DRG*_ = BSE concentration in dorsal root ganglia; *I*_*I&M*_ = BSE infectivity in intestines and mesenteries; *P*_*sc*_ = probability of incomplete removal of spinal cord; *R*_*rend*_ = reduction of BSE infectivity by rendering; *Depth* = depth at which frass or manure is applied; *F*_*soil*_ = fraction of soil ingested with grass or forage crops.

The what-if scenarios provided more insight into the impact of modeling assumptions on the results and included some worst-case scenarios. Most of the what-if scenarios challenging the assumptions on the exposure due to application of frass and manure on grassland or forage crops (WI-13 to WI-16) did not affect the overall exposure of cattle to BSE infectivity (*I*_*r, s*_) (Figure 6, Supplementary Table 3). Assumptions on the exposure via calf milk replacer (WI-18 to WI-21) only affected the overall exposure when it was assumed that all infectivity is retained in fish (WI-18) or when a worst-case scenario was assumed in which all insect meal was used in aquafeed and all fishmeal in calf milk replacer (WI-21). Similarly, the worst-case scenario in which all insect meal is used in pig or poultry feed and all pig and poultry manure used to fertilize grassland (WI-17) resulted in an increased overall exposure of cattle. Assumptions on the infectivity in insect meal slightly affected the estimated overall exposure of cattle to BSE infectivity (WI-6 to WI-9), where especially a lower accumulation of infectivity in insects resulted in a lower overall exposure for most substrate-insect combinations (WI-7, WI-8). Assumptions on the infectivity in the bovine carcass and on cross-contamination of ruminant feed had most impact on model results. Whereas, our assumptions on BSE infectivity in edible tissue (WI-1 and WI-2) did not affect model results, the assumptions on the contribution of the DRG to the total infectivity in edible products and category 2 meat meal made a big difference. Assuming that the BSE concentration in DRG is equal to SRM (WI-3) resulted in a 1.2 log_10_ higher overall exposure of cattle. Assuming removal of DRG similar to what is done in controlled risk countries (WI-4) resulted in a 1.8 to 2.0 log_10_ lower overall exposure. Assuming no removal of SRM at all (WI-5) resulted in a 2.5 to 2.6 log_10_ higher overall exposure. A lower level of cross-contamination of ruminant feed at the feed mill, during transport, and at the farm level (WI-10) resulted in a reduced overall exposure of cattle to BSE infectivity, whereas a higher level of cross-contamination increased the overall exposure of cattle to BSE infectivity (WI-11). Assuming a 100% cross-contamination due to fraud, mislabeling or accidental access of cattle to non-ruminant feed at farm level (WI-12) resulted in a 2.1 to 2.9 log_10_ higher exposure of cattle to BSE infectivity. Nevertheless, the overall exposure was still far below the level of 1 CoID_50_, even when considering the 97.5 percentile values for all substrate-insect combinations (Figure 6, Supplementary Table 3). Some of the what-if scenarios also resulted in a different relative contribution of the exposure pathways to the BSE risk. The most striking examples are WI-7, where all BSE infectivity at harvest is assumed in frass rather than insects; WI-11 and WI-12 where there is a higher cross-contamination of feed assumed; and WI-18 and WI-21 that both resulted in a relatively high contribution of calf milk replacer to the BSE risk (Supplementary Figure 1).

**Figure 6:**
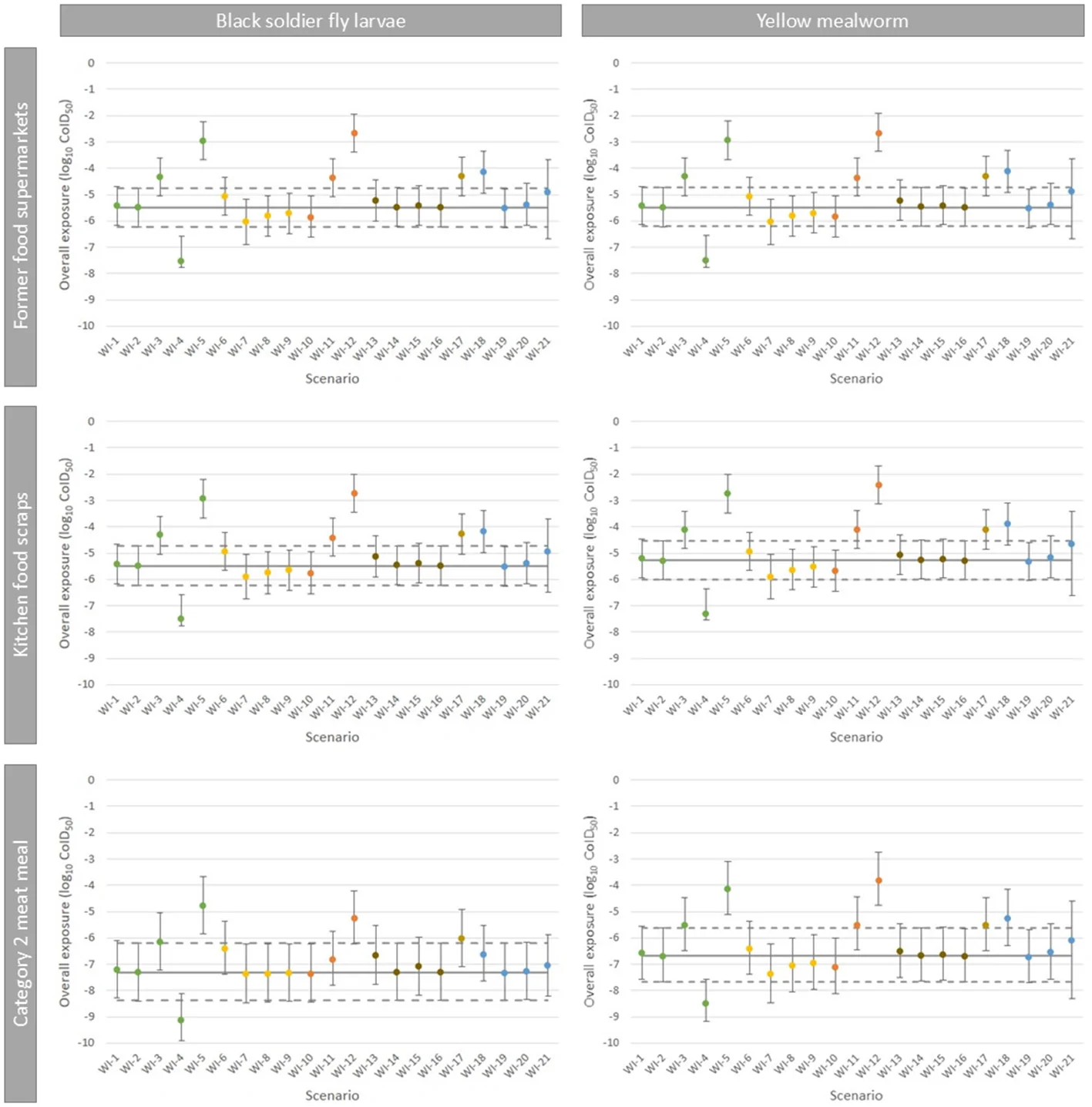
Median values and 95% uncertainty interval of the estimated overall exposure (log10 CoID50) of cattle to BSE infectivity in each of the what-if scenarios for the different substrate-insect combinations. The median exposure of the baseline model results is indicated by the grey solid line; the dotted lines indicate the 95% uncertainty interval of the baseline model results. What-if scenarios challenged assumptions on **(a)** the infectivity in the bovine carcass (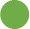); **(b)** infectivity in insect meal (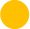); **(c)** exposure due to cross-contamination of feed (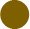); **(d)** exposure due to application of frass and manure on grassland (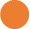); **(e)** a combination of c and d (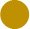); and **(f)** exposure via calf milk replacer (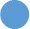). A more detailed description of the what-if scenarios is given in Table 2.

## 4 Discussion

The estimated risk of new BSE infections resulting from insect rearing on residual streams is very low with an expected median probability that < 1 out of 100,000 BSE-infected cows slaughtered at clinical end state would result in one or more new BSE infections via any of the considered exposure routes. This is a conservative estimate, as we mostly used worst-case assumptions on the presence and persistence of prions when no data were available, including the infectivity level in edible tissue, the allocation of prions to insects and frass after insect rearing and the persistence of prions in the gastro-intestinal tract of pigs and poultry and in the environment. Probability distributions used for input parameters mostly represented a mix of biological variability and epistemic uncertainty, as available data were not sufficient to separate these. An exception is the approach used to model cross-contamination at the slaughterhouse due to incomplete removal of SRM or brain tissue embolism caused by stunning when using a penetrating captive bolt gun, for which variability was explicitly modeled using a Bernoulli distribution. Both cross-contamination events had a low probability of occurrence, resulting in a skewed uncertainty distribution for the total exposure of cattle to BSE infectivity (*I*_*i, s*_) as well as the probability of at least one new infection (_*P*_*BSE*_*i, s*_). But even based on 99 percentile values of *I*_*i, s*_ and _*P*_*BSE*_*i, s*_ we can conclude that the risk is very low (Supplementary Table 2). This conclusion is backed by the results of WI-5, where we assumed no removal of SRM at all (Figure 6).

The estimated exposure of cattle to BSE infectivity and the associated risk of new BSE infections differed across the evaluated substrate-insect combinations due to different infectivity levels of the substrates and differences in relative weights of insects and frass at harvest. The latter parameter was based on experimental growth experiments (Meijer et al., 2026; Hoek-van den Hil et al., 2024) and might be variable across different batches of the residual streams. However, where the composition and quality of former food from supermarkets and kitchen food scraps from consumer households is expected to vary across batches, this is likely to be consistent for category 2 meat meal. Model results show that differences in results on exposure are associated more with the substrate than the substrate-insect combination (Figure 3). There are other parameters that also might vary between the insect species considered and therewith impact the levels of exposure for the different substrate-insect combinations, such as the expected market share of insect meal for aquafeed and pig and poultry feed, or the application of frass to grassland and forage crops. These were, however, not considered in the model calculations as we had no data to substantiate different assumptions for BSFL and YMW.

In general, exposure of cattle to BSE infectivity was highest via cross-contamination of ruminant feed, with this route contributing between 61 and 81% for all substrate-insect combinations but BSFL on MM (Figure 4). The different exposure profile for BSFL reared on MM was due to the poor observed growth of BSFL on MM in a pilot experiment (Den Boer and Brugmans, 2022), which resulted in a very low weight fraction of insects at harvest (*F*_*insec*_*t*__*i, s*__, see Table 1). In the model calculations, we therefore assumed that most BSE infectivity was present in the frass rather than the insects for this substrate-insect combination, resulting in a much higher contribution of frass to exposure. The estimated overall exposure of cattle to BSE infectivity was, however, lowest for this substrate-insect combination (Figure 3A). Model results for BSFL on MM have high uncertainty though, as we used an indirect method to estimate the relative weight of insects and frass at harvest. Furthermore, follow-up experiments indicated better growth of the insects on this substrate if supplemented with additional ingredients and water (Borghuis, pers. comm.). Nevertheless, it is not expected that insect growers will use MM for commercial BSFL production if legally permitted as BSFL grow much better on wet substrates such as SM and KF.

The importance of the pathway of cross-contaminated feed was confirmed by the what-if analysis indicating that a higher level of cross-contamination (WI-11 and WI-12) considerably increases the exposure of cattle to BSE infectivity (Figure 6). This stresses the importance of compliance with Regulation (EU) 2021/1372 and to only produce compound feed containing insect meal in feed mills dedicated to the production of feed for aquaculture, pigs or poultry (EC, 2021b). In addition, dedicated transport means and no delivery of compound feed containing insect meal to mixed farms with both ruminants and non-ruminants could further reduce the BSE risk via exposure to contaminated feed. These measures can, however, not prevent fraud or erroneous use of feed, as simulated by WI-12. Yet even in this worst-case scenario, the median total BSE infectivity reaching cattle was less than 0.01 CoID_50_ (Figure 6).

The exposure of cattle to BSE infectivity via calf milk replacer was consistently low for all substrate-insect combinations (Figure 4; Supplementary Table 2). This is also reflected by the low impact of the what-if scenarios challenging the uncertainty on this exposure route, with only the assumption that all BSE infectivity would be retained in the gastro-intestinal tract of fish (WI-18) resulting in a substantially increased risk. The low exposure via calf milk replacer adds to the low BSE risk of insect rearing on the residual streams evaluated, as young animals (up to an age of 6 months) are most susceptible to BSE infection (Arnold and Wilesmith, 2004).

The baseline model results were most sensitive to uncertainty on the concentration of BSE infectivity in specified risk materials (*C*_*SRM*_) and the dorsal root ganglia (*C*_*DRG*_), and the infectivity in intestines and mesenteries (*I*_*I&M*_). In the case of category 2 meat meal substrates, results were also highly sensitivity to the inactivation achieved by rendering (*R*_*rend*_) (Figure 5). Nevertheless, the 97.5% percentile values of the estimated exposure levels in the baseline scenario were < < 1 CoID_50_, indicating that despite these uncertainties the infectivity reaching cattle would not suffice to initiate a new BSE epidemic under the current control measures and given the pathways of exposure considered in the model. The use of residual streams for insect rearing can only result in a new BSE epidemic if the infectivity introduced in the system via a single infected cow would on average result in more than one new BSE infection. The probability that ingestion of 1 CoID_50_ results in infection is 50 %, i.e., on average only 1 out of 2 cattle receiving 1 CoID_50_ will develop BSE. Hence, an exposure < < 1 CoID_50_ is extremely unlikely to result in a new epidemic with the feed-borne exposure route being the only known transmission route for BSE. The worst-case scenarios explored in the what-if analysis, including no removal of SRM at slaughter (WI-5) and 100% cross-contamination of ruminant feed due to fraud, mislabelling or erroneous feeding at the farm level (WI-12), confirmed the low BSE risk of insect rearing on residual streams with a median overall exposure to BSE infectivity < 0.01 CoID_50_ for all substrate-insect combinations and a maximum 97.5 percentile value of 2.0 × 10^−2^ CoID_50_ for YMW reared on KF in case of 100% cross-contamination (Figure 6). It should, however, be kept in mind, that most what-if scenarios explored the effect of a change in only one input parameter at a time. In the extremely unlikely event that all adverse conditions explored in the what-if analysis would occur simultaneously, i.e., no SRM removal (WI-5), retention of prions in insects (WI-6), accidental feeding of insect meal used in pig and poultry feed to cattle (WI-12), higher levels of frass and manure applied to grassland (WI-13 and WI-14), retention of prions in fish (WI-18), and a higher fraction of fishmeal used in CMR (WI-20), the overall exposure of cattle to BSE infectivity reaches much higher levels with a median value of 3.0 CoID_50_ for insects reared on SM, a median value of 4.0 CoID_50_ for insects reared on KF and a median value of 0.13 CoID_50_ for insects reared on MM, with an associated much higher infection risk (Supplementary Figure 2).

The input distributions used to model uncertainty on the infectivity in bovine tissue were based on previous risk assessments performed by EFSA (EFSA, 2018, 2020, 2024b) and De Vos et al. (2024). No quantitative data was available to estimate the infectivity in muscle tissue and evidence on the presence of prions in muscle tissue was inconclusive (Wells et al., 2005; Espinosa et al., 2007; Suardi et al., 2012; EFSA, 2014b). Buschmann and Groschup (2005) concluded that infectivity levels in muscle should at least be 6 log steps lower than in brain tissue. This would implicate an infectivity level slightly above the detection limit of bioassays. We explored the impact of this assumption in the what-if analysis (WI-1) and concluded that it hardly affected the model results. Alternatively, the assumption of infectivity being present just below the detection limit in all edible tissues might have resulted in an overestimate of the risk in the baseline scenario. Assuming no infectivity in muscle tissue at all (WI-2) did, however, not change model results either. On the other hand, assumptions on the infectivity level of the dorsal root ganglia had considerable impact on the estimated exposure of cattle to BSE infectivity. In our baseline calculations, we used the estimate given by EFSA (2018) as this was supported by data from a pathogenesis study estimating cattle tissue infectivity (Arnold et al., 2009). EFSA (2020, 2024b) did, however, assume infectivity levels in the DRG to equal those in SRM, which is definitely a worst-case assumption (WI-3). Results of scenario WI-4, where removal of SRM is assumed as prescribed for countries with a controlled or undetermined BSE risk, were used to cross-validate our assumptions on the concentration of BSE infectivity in meat with estimates provided by EFSA (2018) that also assumed removal of SRM. Where EFSA (2018) reported 1 CoID_50_ (95% uncertainty interval 0.002–8) for the total infectivity in the category 3 animal byproducts from an infected but non-detected animal, we estimated a mean total infectivity of 0.79 CoID_50_ (95% uncertainty interval 0.08–1.3) in meat for scenario WI-4, which is in the same order of magnitude.

The main uncertainty in this study was, however, the fate of prions during insect rearing, i.e., the carry-over of prions from substrate to insects. Theoretically, there is three scenarios for the fate of BSE prions in insects: insects could (partially) inactivate the prions, resulting in a loss of infectivity during rearing; insects could act as a transmission medium of prions (mechanical vector), resulting in an equal level of infectivity before and after rearing; and insects could amplify the prions (biological vector), resulting in an increase of infectivity during rearing. The latter option was deemed unlikely as insects do not have natural prion proteins (Post et al., 1999; Bujdoso et al., 2023; Benestad et al., 2024). In the baseline model calculations, we therefore assumed relay of prions without accumulation in the insects, i.e., we assumed an equal distribution across insects and frass based on relative weights. If accumulation in insects would occur, mimicked by scenario WI-6 assuming 100% of prions being retained in the insects, the expected exposure of cattle to BSE infectivity is 0.3-0.9 log_10_ CoID_50_ higher (Figure 6). We considered relay of prions already a worst-case scenario though, as the quantity of prions in the insects is likely to be lower than the amount of prions they encountered (EFSA, 2015; Benestad et al., 2024). Assuming only prions in the guts of insects (WI-8) or all prions in frass (WI-7) resulted in a 0.3-0.4 log_10_ CoID_50_ and 0.4-0.7 log_10_ CoID_50_ lower exposure, respectively (Figure 6) for all substrate-insect combinations but BSFL reared on MM. It should be noted that the effect of these what-if scenarios was different for the latter combination, because of the assumed low infectivity level in the insects in the baseline scenario due to the poor growth performance of BSFL on MM.

There is hardly any data available regarding prions in insects farmed for feed and food (Vandeweyer et al., 2021). However, there are reports on isolation of scrapie from parasitic flies (*Sarcophaga carnaria, Oestrus ovis*) and hay mites, as well as the transmission of scrapie to hamsters or mice upon ingestion of these flies or mites indicating that insects could indeed act as a mechanical vector of prions (Wisniewski et al., 1996; Post et al., 1999; Corona et al., 2006; EFSA, 2015; Anonymous, 2019). A recent study by Benestad et al. (2024) was the first to investigate the potential role of BSFL as a vector of prion disease by exposing BSFL to brain homogenates of scrapie-positive sheep. They were able to detect prions in BSFL by PMCA (protein misfolding cyclic amplification) not only after a single day of feeding on the contaminated substrate, but also after several days of fasting. Using PMCA might have led to an overestimation of results, as this detection method is 10^2^ to 10^5^-fold more sensitive than bioassays in rodents, which are considered the golden standard for assessing prion infectivity potential (Saá et al., 2006; Makarava et al., 2012; Moudjou et al., 2013). The primary objective of the study of Benestad et al. (2024) was to obtain evidence of prions being retained in BSFL; the experimental set up did not mimic a real-life scenario of contaminated residual streams and no attempt was made to quantify the prions. Further challenge studies are needed to determine the epidemiological implications of prions retained in the insects, including the potential of subsequent transmission to susceptible ruminants upon oral exposure. Results of such studies might require a reiteration of this risk assessment.

Studies on the fate of ruminant DNA in insects could potentially be used as a proxy to estimate the carry-over of prions from the substrate to the insects. No conclusions can, however, be inferred from these studies as the relationship between the carry-over of ruminant DNA and prion proteins has not been investigated. Hoffmans et al. (2024) performed DNA analysis for both substrate and BSFL when the insects were reared on fast food waste. Ruminant DNA appeared to be present in both the residual stream tested (fast food) and the BSFL. Appel et al. (2026) came to similar conclusions when rearing BSFL on fast food waste. However, no ruminant DNA was detected in the BSFL after a 7-day rearing period on catering waste or kitchen food scraps. Not unexpectedly, ruminant DNA was present in the frass of insects reared on fast food and catering waste; the frass of insects reared on kitchen food scraps could not be tested due to inhibition of the qPCR assay. Appel et al. (2026) also showed that ruminant DNA was cleared from the insects after a 2-4 day recovery period on a plant-based substrate, which could be considered as a risk mitigation strategy.

In the model, a homogenous distribution of BSE infectivity in both the contaminated tissues of an infected animal, and the different products produced along the pathway from slaughtered cow to exposure of cattle was assumed. With this, we ignored the possibility of clumping of prions, which could have resulted in either lower or higher infectivity levels in the different steps of the model. The impact of this assumption was not explicitly addressed in the what-if analysis. However, some of the scenarios accounted for a single destination of insect meal (WI-17, WI-21) and as such mimicked the effect of clustering of infectivity resulting in a slightly higher exposure of cattle (Figure 6). Furthermore, clumping of infectivity could also result in a higher probability of infection of individual animals, if it results in exposure to a higher infectivity load. This is especially important if a minimum infectious dose is required to result in infection, something which is currently not known (EFSA, 2020). The exponential dose-response model (Equation 14) that we used to estimate the probability of infection is a worst-case approach, as it assumes that there is no threshold dose for prions (Gale, 1998). The final dose of BSE infectivity to which individual animals will be exposed is likely to be much lower than the estimated BSE infectivity reaching cattle via each of the individual exposure routes. If the resulting individual dose is below the minimum infectious dose, exposure will not result in infection at all.

The model calculations did not differentiate between classical BSE (C-BSE) and atypical BSE strains (H-BSE and L-BSE), as we did not have quantitative data to distinguish between these different BSE strains. Possible differences between the strains would be the distribution of infectivity across bovine tissues and the transmissibility by oral uptake (EFSA, 2014b). Presence of prions in muscle tissue has been found mostly in cattle infected with the atypical forms of BSE (Konold et al., 2012a; Suardi et al., 2012). However, the presence of prions in muscle tissue seems to be less likely in asymptomatic cattle (Suardi et al., 2012; Okada et al., 2014). The scenario of BSE-infected cattle being slaughtered and declared fit for human consumption is most likely for cattle still incubating the disease and not showing clinical symptoms yet, supporting our assumption of infectivity levels in muscle tissue at or below the detection limit of bioassays for all BSE strains. Feeding of infected material is considered an important route of transmission for C- BSE (EFSA, 2018; Kumagai et al., 2019; Alarcon et al., 2023), whereas the transmission routes of H-BSE and L-BSE have not been elucidated yet, if these strains are being transmitted at all (Dudas and Czub, 2017). So far, epidemiological observations do not support transmission of the atypical BSE strains and the hypothesis is that infections occur spontaneously (CFSPH, 2016; Kumagai et al., 2019; WOAH, 2024a). Also, the oral dose needed for successful transmission of atypical BSE is higher compared to classical BSE (Okada et al., 2017; Kumagai et al., 2019). Transmission of these strains by feed can, however, not be completely ruled out when TSE regulations, such as the feed ban, species-to-species ban and removal of specified risk material, would be lifted. Therefore, we argue that based on the current knowledge and from a risk-averse perspective, the BSE risk of feeding insects on residual streams containing animal proteins should be considered equal for C-BSE, H-BSE and L-BSE.

The risk assessment in this study is based on the infectivity ending up in residual streams when a single BSE-infected cow is slaughtered undetected and thus does not provide the annual or population-level risk for the EU as a whole Over the last decade, very few BSE-infected cattle have been detected annually in the EU (WOAH, 2024b; EFSA, 2024a). The majority of these infections were typed as H-BSE and L-BSE; no C-BSE infections were detected in the EU since 2019, although the United Kingdom reported on an additional three C-BSE cases in 2021, 2024 and 2026 (APHA, 2021, 2024; WOAH, 2026). Based on 2015 surveillance data, the number of BSE-infected cattle (C-BSE, H-BSE and L-BSE combined) that is slaughtered undetected each year in the EU was estimated at 11.4 (95 % uncertainty interval 3.6 – 19.8) (EFSA, 2018). Given the low, but stable level of BSE infections detected in Europe during the period 2015–2023, we considered this estimate representative for the current situation. Considering that the total number of adult cattle (> 2 years) in the EU was over 36 million in 2023 (EFSA, 2024a), the probability of a residual stream being infected at all due to the slaughter of an undetected BSE-infected cow is thus extremely low. The probability of BSE-infected cattle being slaughtered undetected also depends on the surveillance in place. The risk estimate of this study assumes no other relaxations of the TSE regulation than the use of residual streams containing animal proteins as a substrate for insect rearing. Additional relaxations could increase the risk of new BSE infections, although the what-if analysis indicates that the exposure of cattle to BSE infectivity is likely to be < 1 CoID_50_ even when SRM removal would be abandoned (WI-12). Nevertheless, it is advised to reiterate this risk assessment whenever legislation or other conditions or circumstances would change. Similarly, future changes in the market share of insect meal in aquafeed or pig and poultry feed might affect the estimated BSE risk, as indicated by scenarios WI-17 and WI-21 of the what-if analysis. Close monitoring of the BSE risk is also warranted if circular economy practices scale up and the volume of waste stream processing is increased. A breach of the safety guarantees, e.g., at the slaughterhouse or in the feed mill, could result in an increased exposure of cattle to BSE infectivity. Timely detection of new BSE infections is challenging given the long incubation period in cattle, as a result of which new infections could go unnoticed for a long time.

## 5 Conclusion

Based on the results of this risk assessment we conclude that the risk of new BSE infections due to insect feeding on residual streams containing animal proteins is very low under the current EU control system, provided that insects cannot act as a biological vector of prions. The assessment was based on a stochastic risk model estimating the exposure of cattle to BSE-infectivity via insect meal when a single BSE-infected cow escapes detection. We mostly used worst-case assumptions when no data were available to estimate the presence and persistence of prions resulting in a conservative estimate of the risk, arguing that we err on the safe side if risks are low even under those conditions. This is especially true for the infectivity level in edible tissue, the carry-over of BSE infectivity by insects and frass and the persistence of prions in the gastro-intestinal tract of pigs and poultry and in the environment. Nevertheless, the estimated exposure of cattle to BSE infectivity was < 1 CoID_50_ in all scenarios modeled, including those assuming no removal of SRM or 100% cross-contamination of ruminant feed (resulting in all insect meal used for pig and poultry feed being fed to ruminants). The model was applied to three specific residual streams and limited to two insect species, and although all of these showed a similarly low risk, we recommend to update and run the model for every new residual stream or insect species. Likewise, model calculations need to be reiterated when new evidence on the fate of prions in insects would become available, or when changes in legislation would affect the estimated risk. The results of this study indicate an equally low risk for insect rearing on residual streams as for the feeding of pig PAP to poultry and poultry PAP to pigs (EFSA, 2018), or the use of ruminant collagen and gelatine in non-ruminant feed (EFSA, 2020, 2024b), both of which were recently legalized in the EC (2021b). Similarly, results of this study could contribute to the re-evaluation of the EU regulatory framework for insect production and open the way to insect rearing on residual streams containing animal proteins. It should, however, be kept in mind that a full appraisal of the veterinary risk of insect rearing on residual streams also requires an assessment of the transmission risk of other animal diseases such as foot-and-mouth disease and African swine fever.

## Data Availability

The original contributions presented in the study are included in the article/Supplementary material, further inquiries can be directed to the corresponding author.

## References

[B1] Abd Manan F. Yeoh Y. K. Chai T. T. Wong F. C. (2024). Unlocking the potential of black soldier fly frass as a sustainable organic fertilizer: a review of recent studies. J. Environ. Manage. 367:121997. doi: 10.1016/j.jenvman.2024.121997 39111002

[B2] Agrimatie (2024). Gewasopbrengsten – Melkveehouderij. Available online at: https://agrimatie.nl/PublicatiePage.aspx?subpubID=2523andsectorID=2245andthemaID=2754andindicatorID=3551. (Accessed on November 13, 2024)

[B3] Alao B. O. Falowo A. B. Chulayo A. Muchenje V. (2017). The potential of animal by-products in food systems: production, prospects and challenges. Sustainability 9:1089. doi: 10.3390/su9071089

[B4] Alarcon P. Wall B. Barnes K. Arnold M. Rajanayagam B. Guitian J. (2023). Classical BSE in Great Britain: review of its epidemic, risk factors, policy and impact. Food Control 146:109490. doi: 10.1016/j.foodcont.2022.109490

[B5] Alltech Coppens (2023). Regenboogforel. Wat is Belangrijk om te Weten? Available online at: https://www.alltechcoppens.com/uploads/NL-2022-05-Trout-species-tool.pdf?v=1676548349 (Accessed November 14, 2024).

[B6] Anderson K. N. Woiwode R. Hamilton E. M. Kirk A. A. Cowell M. J. Reyes A. A. . (2025). Relationship between captive bolt placement, tissue parameters, and brain contact in cadaver heads from culled Holstein cows >30 mo of age. J. Dairy Sci. 108, 11279–11302. doi: 10.3168/jds.2025-26338 40451587

[B7] Anonymous (2019). Advice on Animal and Public Health Risks of Insects Reared on Former Foodstuffs As Raw Material for Animal Feed. Netherlands Food and Consumer Product Safety Authority, Utrecht, the Netherlands. Available online at: https://english.nvwa.nl/documents/food/safety/documents/advice-on-animal-and-public-health-risks-of-insects-reared-on-former-foodstuffs-as-raw-material-for-animal-feed. (Accessed on December 5, 2024)

[B8] APHA (2021). Report on the epidemiological investigation of a single BSE case in Somerset, England (RBSE 2021:00002). Available online at: https://assets.publishing.service.gov.uk/media/61ba166b8fa8f50384489d16/Report_on_the_epidemiological_investigation_of_a_single_BSE_case_in_Somerset.pdf. (Accessed on September 3, 2026).

[B9] APHA (2024). Report on the Epidemiological Investigation of a BSE Case in Scotland (RBSE24_00003). Available online at: https://assets.publishing.service.gov.uk/media/67225af53ce5634f5f6ef578/bse-epidemiological-report-scotland-2024.pdf. (Accessed on September 3, 2026).

[B10] Appel M. J. Van Rozen K. Van Wikselaar P. G. Prevo-Keijbets E. van Vuure C. ten Holte M. . (2026). Safe use of residual streams for animal feed: hazard inventory and tracing banned animal material in Black Soldier Fly Larvae. J. Insects Food Feed.

[B11] Arnold M. E. Hawkins S. A. Green R. Dexter I. Wells G. A. (2009). Pathogenesis of experimental bovine spongiform encephalopathy (BSE): estimation of tissue infectivity according to incubation period. Vet. Res. 40:8. doi: 10.1051/vetres:2008046 PMC2695012 18950589

[B12] Arnold M. E. Simons R. R. L. Hope J. Gibbens N. Adkin A. L. (2017). Is there a decline in bovine spongiform encephalopathy cases born after reinforced feed bans? A modelling study in EU member states. Epidemiol. Infect. 145, 2280–2286. doi: 10.1017/S0950268817001236 PMC9148812 28625169

[B13] Arnold M. E. Wilesmith J. W. (2004). Estimation of the age-dependent risk of infection to BSE of dairy cattle in Great Britain. Prev. Vet. Med. 66, 35–47. doi: 10.1016/j.prevetmed.2004.07.007 15579333

[B14] Aspevik T. Oterhals Å. Rønning S. B. Altintzoglou T. Wubshet S. G. Gildberg A. . (2017). Valorization of proteins from co- and by-products from the fish and meat industry. Top. Curr. Chem. 2018:53. doi: 10.1007/s41061-017-0143-6 28466455

[B15] Athanassiou C. G. Coudron C. L. Deruytter D. Rumbos C. I. Gasco L. Gai F. . (2024). A decade of advances in black soldier fly research: from genetics to sustainability. J. Insects Food Feed 11, 219–246. doi: 10.1163/23524588-00001122

[B16] Ball M. E. E. Wright L. P. Wilson K. Richmond H. Cummings R. Smyth S. . (2024). The Nutrient content of litter and manure from different poultry systems—updating and establishing the nutrient profile. Sustainability 16:6633. doi: 10.3390/su16156633

[B17] Ballarin C. Povinelli M. Granato A. Panin M. Corain L. Peruffo A. . (2016). The brain of the domestic bos taurus: weight, encephalization and cerebellar quotients, and comparison with other domestic and wild cetartiodactyla. PloS ONE 11:e0154580. doi: 10.1371/journal.pone.0154580 PMC4851379 27128674

[B18] Beltran-Alcrudo D. Falco J. R. Raizman E. Dietze K. (2019). Transboundary spread of pig diseases: the role of international trade and travel. BMC Vet. Res. 15:64. doi: 10.1186/s12917-019-1800-5 PMC6387505 30795759

[B19] Benestad S. L. Tran L. Malzahn A. M. Liland N. S. Belghit I. Hagemann A. (2024). Retention of prions in the polychaete Hediste diversicolor and black soldier fly, *Hermetia illucens*, larvae after short-term experimental immersion and feeding with brain homogenate from scrapie infected sheep. Heliyon 10:e34848. doi: 10.1016/j.heliyon.2024.e34848 PMC11336280 39170463

[B20] Blanken K. Evers A. Groeneveld I. Niyonsaba H. Ouweltjes W. Verkaik J. . (2022). KWIN 2022-2023. Kwantitatieve Informatie Veehouderij. Handboek 46. Wageningen University and Research. (in Dutch)

[B21] Broeckx L. Frooninckx L. Slegers L. Berrens S. Noyens I. Goossens S. . (2021). Growth of Black Soldier Fly Larvae Reared on Organic Side-Streams. Sustainability 13:12953. doi: 10.3390/su132312953

[B22] Bruno D. Orlando M. Testa E. Carnevale Miino M. Pesaro G. Miceli M. . (2025). Valorization of organic waste through black soldier fly: on the way of a real circular bioeconomy process. Waste Manag. 191, 123–134. doi: 10.1016/j.wasman.2024.10.030 39531740

[B23] Bujdoso R. Smith A. Fleck O. Spiropoulos J. Andréoletti O. Thackray A. M. (2023). Prion disease modelled in Drosophila. Cell Tissue Res. 392, 47–62. doi: 10.1007/s00441-022-03586-0 PMC10113284 35092497

[B24] Buschmann A. Groschup M. H. (2005). Highly bovine spongiform encephalopathy-sensitive transgenic mice confirm the essential restriction of infectivity to the nervous system in clinically diseased cattle. J. Infect. Dis. 192, 934–942. doi: 10.1086/431602 16088845

[B25] CFSPH (2016). Bovine Spongiform Encephalopathy. Technical Factsheet. The Center For Food Security and Public Health. Iowa State University. Available online at: https://www.cfsph.iastate.edu/Factsheets/pdfs/bovine_spongiform_encephalopathy.pdf. (Accessed on July 2, 2024)

[B26] Coore R. R. Love S. McKinstry J. L. Weaver H. R. Philips A. Hillman T. . (2005). Brain tissue fragments in jugular vein blood of cattle stunned by use of penetrating or nonpenetrating captive bolt guns. J. Food Prot. 68, 882–884. doi: 10.4315/0362-028X-68.4.882 15830690

[B27] Corona C. Martucci F. Iulini B. Mazza M. Acutis P. L. Porcario C. . (2006). Could *Oestrus ovis* act as vector for scrapie? Abstracts Prion Torino 4–6, 133.

[B28] Cummins E. Adkin A. (2007). Exposure assessment of TSEs from the landspreading of meat and bone meal. Risk Anal. 27, 1179–1202. doi: 10.1111/j.1539-6924.2007.00953.x 18076490

[B29] Dagevos H. Verhoog D. Van Horne P. Hoste R. (2023). Vleesconsumptie Per Hoofd Van de Bevolking in Nederland, 2005-2022. Wageningen Economic Research, Wageningen University and Research. Nota 2023-139. Available online at: https://edepot.wur.nl/638735. (Accessed October 18, 2024).

[B30] Davies G. (2002). The foot and mouth disease (FMD) epidemic in the United Kingdom 2001. Comp. Immunol. Microbiol. Infect. Dis. 25, 331–343. doi: 10.1016/S0147-9571(02)00030-9 12365809

[B31] De Vos C. J. Antonis A. F. G. Sturme M. H. J. Appel M. (2024). Risk of BSE transmission when fishmeal derived from fish fed bovine spray-dried red blood cells is included in calf milk replacers. Microb Risk Anal. 27-28, 100323. doi: 10.1016/j.mran.2024.100323

[B32] De Vos C. J. Heres L. (2009). The BSE risk of processing meat and bone meal in nonruminant feed: a quantitative assessment for the Netherlands. Risk Anal. 29, 541–557. doi: 10.1111/j.1539-6924.2008.01177.x 19144064

[B33] Den Boer S. Brugmans F. (2022). Can black soldier fly larvae safely grow on unapproved residual streams? (BSc Thesis). ['s-Hertogenbosch, The Netherlands]: HAS green academy

[B34] Dixon L. K. Sun H. Roberts H. (2019). African swine fever. Antiviral Res. 165, 34–41. doi: 10.1016/j.antiviral.2019.02.018 30836106

[B35] Doughty K. H. Middleton J. A. Salini M. J. Kragt M. E. Partridge J. C. Hemmi J. M. . (2024). Food for thought: valuable bioproduction pathways emerge in a circular food production model. Clean. Circ. Bioecon. 9:100102. doi: 10.1016/j.clcb.2024.100102

[B36] Ducrot C. Sala C. Ru G. de Koeijer A. Sheridan H. Saegerman C. . (2010). Modelling BSE trend over time in Europe, a risk assessment perspective. Eur. J. Epidemiol. 25, 411–419. doi: 10.1007/s10654-010-9455-3 20386960

[B37] Dudas S. Czub S. (2017). Atypical BSE: current knowledge and knowledge gaps. Food Saf. 5, 10–13. doi: 10.14252/foodsafetyfscj.2016028 PMC6989174 32231923

[B38] EC (2001). Regulation (EC) No 999/2001 of the European Parliament and of the Council of 22 May 2001 laying down rules for the prevention, control and eradication of certain transmissible spongiform encephalopathies. Off. J. Eur. Commun. L 147, 1–40.

[B39] EC (2008). Commission Regulation (EC) No 956/2008 of 29 September 2008 amending Annex IV to Regulation (EC) No 999/2001 of the European Parliament and of the Council laying down rules for the prevention, control and eradication of certain transmissible spongiform encephalopathies. Off. J. Eur. Commun. 260, 8–11.

[B40] EC (2009). Regulation (EC) No 1069/2009 of the European Parliament and of the Council of 21 October 2009 laying down health rules as regards animal by-products and derived products not intended for human consumption and repealing Regulation (EC) No 1774/2002 (Animal by-products Regulation). Off. J. Eur. Commun. 300, 1–33.

[B41] EC (2011). Commission Regulation (EU) No 142/2011 of 25 February 2011 implementing Regulation (EC) No 1069/2009 of the European Parliament and of the Council laying down health rules as regards animal by-products and derived products not intended for human consumption and implementing Council Directive 97/78/EC as regards certain samples and items exempt from veterinary checks at the border under that Directive. Off. J. Eur. Union L 54, 1–54.

[B42] EC (2017). Commission Regulation (EU) 2017/893 of 24 May 2017 amending Annexes I and IV to Regulation (EC) No 999/2001 of the European Parliament and of the Council and Annexes X, XIV and XV to Commission Regulation (EU) No 142/2011 as regards the provisions on processed animal protein. Off. J. Eur. Union L 138, 92–116.

[B43] EC (2021a). Commission Regulation (EU) 2021/1925 of 5 November 2021 amending certain Annexes to Regulation (EU) No 142/2011 as regards the requirements for placing on the market of certain insect products and the adaptation of a containment method. Off. J. Eur. Union L 393, 4–8.

[B44] EC (2021b). Commission Regulation (EU) 2021/1372 of 17 August 2021 amending Annex IV to Regulation (EC) No 999/2001 of the European Parliament and of the Council as regards the prohibition to feed non-ruminant farmed animals, other than fur animals, with protein derived from animals. Off. J. Eur. Union L 295, 1–17.

[B45] EC (2022). Commission Implementing Decision (EU) 2022/1377 of 4 August 2022 amending the Annex to Decision 2007/453/EC as regards the BSE status of France (notified under document C(2022) 5507). Off. J. Eur. Union L 206, 51–54.

[B46] EFSA (2004). Report of the EFSA working group on BSE risk from dissemination of brain particles in blood and carcass. Question N?EFSA-Q-2003-122. Annex EFSA J. 123, 1–4 on the opinion of the European Food Safety Authority on BSE risk from dissemination of brain particles in blood and carcass following stunning. EFSA J. 123, 1-4. doi: 10.2903/j.efsa.2004.123

[B47] EFSA (2007). Opinion of the scientific panel on biological hazards on the request from the European Commission on a Transmissible Spongiform Encephalopathy risk assessment of the use of bovine spray dried red cells in feeds for fish, in consideration of a report produced by the European Animal Protein Association. EFSA J. 596, 1–45. doi: 10.2903/j.efsa.2007.596

[B48] EFSA (2014a). Scientific Opinion on BSE risk in bovine intestines and mesentery. EFSA J. 12:3554. doi: 10.2903/j.efsa.2014.3554

[B49] EFSA (2014b). Protocol for further laboratory investigations into the distribution of infectivity of Atypical BSE. EFSA J. 12:3798. doi: 10.2903/j.efsa.2014.3798

[B50] EFSA (2015). Scientific Opinion on a risk profile related to production and consumption of insects as food and feed. EFSA J. 13:4257. doi: 10.2903/j.efsa.2015.4257

[B51] EFSA (2018). Scientific opinion on an updated quantitative risk assessment (QRA) of the BSE risk posed by processed animal protein (PAP). EFSA J. 16:5314. doi: 10.2903/j.efsa.2018.5314 PMC7009728 32625957

[B52] EFSA (2020). Scientific Opinion on the potential BSE risk posed by the use of ruminant collagen and gelatine in feed for non-ruminant farmed animals. EFSA J. 18:6267. doi: 10.2903/j.efsa.2020.6267 PMC7592076 33144887

[B53] EFSA (2024a). The European Union summary report on surveillance for the presence of transmissible spongiform encephalopathies (TSE) in 2023. EFSA J. 22:e9097. doi: 10.2903/j.efsa.2024.9097 PMC11603091 39610400

[B54] EFSA (2024b). BSE risk posed by ruminant collagen and gelatine derived from bones. EFSA J. 22:e8883. doi: 10.2903/j.efsa.2024.8883 PMC11249823 39015303

[B55] Espinosa J. C. Morales M. Castilla J. Rogers M. Torres J. M. (2007). Progression of prion infectivity in asymptomatic cattle after oral bovine spongiform encephalopathy challenge. J. Gen. Virol. 88, 1379–1383. doi: 10.1099/vir.0.82647-0 17374785

[B56] Eurostat (2024). Main Livestock Indicators by NUTS 2 Region. Available online at: https://ec.europa.eu/eurostat/databrowser/view/ef_lsk_main__custom_13396737/default/table?lang=en. (Accessed on October 21, 2024)

[B57] FAO (1986). The Production of Fish Meal and oil. FAO Fisheries Technical Paper 142. Food and Agriculture Organization of the United Nations, Rome. Available online at: https://www.fao.org/3/X6899e/X6899E00.HTM. (Accessed on November 14, 2024).

[B58] Franz M. Eiden M. Balkema-Buschmann A. Greenlee J. Schatzl H. Fast C. . (2012). Detection of PrP(Sc) in peripheral tissues of clinically affected cattle after oral challenge with bovine spongiform encephalopathy. J. Gen. Virol. 93, 2740–2748. doi: 10.1099/vir.0.044578-0 22915695

[B59] Gale P. (1998). Quantitative BSE risk assessment: relating exposures to risk. Lett. Appl. Microbiol. 27, 239–242. doi: 10.1046/j.1472-765X.1998.00449.x 9830136

[B60] Haas C. N. Rose J. B. Gerba C. P. (1999). Quantitative Microbial Risk Assessment. John New York, NY: Wiley and Sons, Inc.,

[B61] Hicks T. M. Verbeek C. J. R. (2016). “Meat industry protein by-products: sources and characteristics,” in Protein Byproducts Transformation from Environmental Burden Into Value-Added Products, ed. Dhillon G.S. (London: Academic Press, Elsevier), 37–61. doi: 10.1016/B978-0-12-802391-4.00003-3

[B62] Hoek-van den Hil E. F. Meijer N. P. Van Rozen K. Elissen H. Van Wikselaar P. G. Brust H. . (2024). Safety of black soldier fly (Hermetia illucens) larvae reared on waste streams of animal and vegetal origin and manure. J. Insects Food Feed 10, 771–783. doi: 10.1163/23524588-20230080

[B63] Hoffmans Y. Veldkamp T. Meijer N. P. Brust G. M. H. van der Schans M. G. M. Prins T. W. . (2024). Can black soldier fly larvae (Hermetia illucens) be reared on waste streams for food and feed? – A safety perspective. J. Insects Food Feed 10, 1211–1221. doi: 10.1163/23524588-20230169

[B64] Houben D. Daoulas G. Faucon M. P. Dulaurent A. M. (2020). Potential use of mealworm frass as a fertilizer: impact on crop growth and soil properties. Sci. Rep. 10:4659. doi: 10.1038/s41598-020-61765-x PMC7069999 32170150

[B65] Ingrosso L. Novoa B. Valle A. Z. Cardone F. Aranguren R. Sbriccoli M. . (2006). Scrapie infectivity is quickly cleared in tissues of orally-infected farmed fish. BMC Vet. Res. 2:21. doi: 10.1186/1746-6148-2-21 PMC1513558 16776828

[B66] IPIFF (2023). Overview of the European Insect Feed Market. International Platform of Insects for Food and Feed. Available online at: https://ipiff.org/wp-content/uploads/2024/02/ipiff_market_factsheet_2023-MODIFIED-2-1.pdf. (Accessed on June 30, 2026)

[B67] Köninger J. Lugato E. Panagos P. Kochupillai M. Orgiazzi A. Briones M. J. I. (2021). Manure management and soil biodiversity: towards more sustainable food systems in the EU. Agric. Syst. 194:103251. doi: 10.1016/j.agsy.2021.103251

[B68] Konold T. Arnold M. E. Austin A. R. Cawthraw S. Hawkins S. A. Stack M. J. . (2012b). Bovine spongiform encephalopathy: the effect of oral exposure dose on attack rate and incubation period in cattle - an update. BMC Res. Notes 8:22. doi: 10.1186/1756-0500-5-674 PMC3543162 23217206

[B69] Konold T. Bone G. E. Clifford D. Chaplin M. J. Cawthraw S. Stack M. J. . (2012a). Experimental H-type and L-type bovine spongiform encephalopathy in cattle: observation of two clinical syndromes and diagnostic challenges. BMC Vet. Res. 8:22. doi: 10.1186/1746-6148-8-22 PMC3378435 22401036

[B70] Kumagai S. Daikai T. Onodera T. (2019). Bovine spongiform encephalopathy – a review from the perspective of food safety. Food Saf. 7, 21–47. doi: 10.14252/foodsafetyfscj.2018009 PMC6978881 31998585

[B71] Liceaga A. M. (2021). Processing insects for use in the food and feed industry. Curr. Opin. Insect Sci. 48, 32–36. doi: 10.1016/j.cois.2021.08.002 34455091

[B72] Lumivero (2025). @RISK, Probabilistic Risk Analysis in Excel. Available online at: https://lumivero.com/products/at-risk/. (Accessed on March 5, 2025).

[B73] Makarava N. Savtchenko R. Alexeeva I. Rohwer R. G. Baskakov I. V. (2012). Fast and ultrasensitive method for quantitating prion infectivity titre. Nat. Commun. 3:741. doi: 10.1038/ncomms1730 PMC3518416 22415832

[B74] Marín-Moreno A. Fernández-Borges N. Espinosa J. C. Andréoletti O. Torres J. M. (2017). Transmission and Replication of Prions. Prog. Mol. Biol. Transl. Sci. 150, 181–201. doi: 10.1016/bs.pmbts.2017.06.014 28838661

[B75] Meijer N. Van Raamsdonk L. W. D. Gerrits E. W. J. Appel M. J. (2023). The use of animal by-products in a circular bioeconomy: time for a TSE road map 3? Heliyon 9:e14021. doi: 10.1016/j.heliyon.2023.e14021 PMC10023914 36942230

[B76] Meijer N. Van Zadelhoff K. Dame-Korevaar M. A. Borghuis A. Van Groenestijn J. W. Boonstra J. . (2026). Food and feed safety of *Tenebrio molitor* reared on yet to be legally authorised residual streams in the European Union. J. Insects Food and Feed. 12, 1969–1987 doi: 10.1163/23524588-bja10373

[B77] Melgar-Lalanne G. Hernández-Álvarez A. J. Salinas-Castro A. (2019). Edible insects processing: traditional and innovative technologies. Compr. Rev. Food Sci. Food Saf. 18, 1166–1191. doi: 10.1111/1541-4337.12463 33336989

[B78] Meyer A. M. Meijer N. Hoek-van den Hil E. F. Van der Fels-Klerx H. J. (2021). Chemical food safety hazards of insects reared for food and feed. J. Insects Food Feed 7, 823–831. doi: 10.3920/JIFF2020.0085

[B79] Moudjou M. Sibille P. Fichet G. Reine F. Chapuis J. Herzog L. . (2013). Highly infectious prions generated by a single round of microplate-based protein misfolding cyclic amplification. mBio 5, e00829–e00813. doi: 10.1128/mBio.00829-13 PMC3884057 24381300

[B80] Muscat A. de Olde E. M. Ripoll-Bosch R. Van Zanten H. H. E. Metze T. A. P. Termeer C. J. A. M. . (2021). Principles, drivers and opportunities of a circular bioeconomy. Nat Food 2, 561–566. doi: 10.1038/s43016-021-00340-7 37118163

[B81] Naser El Deen S. van Rozen K. Elissen H. van Wikselaar P. Fodor I. van der Weide R. . (2023). Bioconversion of different waste streams of animal and vegetal origin and manure by black soldier fly larvae *Hermetia illucens* L. (Diptera: Stratiomyidae). Insects 14:204. doi: 10.3390/insects14020204 PMC9968099 36835773

[B82] Ojha S. Bußler S. Schlüter O. K. (2020). Food waste valorisation and circular economy concepts in insect production and processing. Waste Manag. 118, 600–609. doi: 10.1016/j.wasman.2020.09.010 33010691

[B83] Okada H. Iwamaru Y. Imamura M. Miyazawa K. Matsuura Y. Masujin K. . (2017). Oral transmission of L-type bovine spongiform encephalopathy agent among cattle. Emerg. Infect. Dis. 23, 284–287. doi: 10.3201/eid2302.161416 PMC5324790 28098532

[B84] Okada H. Miyazawa K. Fukuda S. Iwamaru Y. Imamura M. Masujin K. . (2014). The presence of disease-associated prion protein in skeletal muscle of cattle infected with classical bovine *spongiform encephalopathy*. J. Vet. Med. Sci. 76, 103–107. doi: 10.1292/jvms.13-0363 PMC3979948 23986118

[B85] Parniakov O. Mikhrovska M. Wiktor A. Alles M. Ristic D. Bogusz R. . (2021). Insect processing for food and feed: a review of drying methods. Dry Technol. 40, 1500–1513. doi: 10.1080/07373937.2021.1962905

[B86] Post K. Riesner D. Walldorf V. Mehlhorn H. (1999). Fly larvae and pupae as vectors for scrapie. Lancet 354, 1969–1970. doi: 10.1016/S0140-6736(99)00469-9 10622303

[B87] Ramantanis S. B. HadŽiosmanović M. Stubičan D. (2005). Preventive measure against possible BSE-hazard: irreversible electrical cattle stunning - a review. Vet. Arh. 75, 83–100.

[B88] Saá P. Castilla J. Soto C. (2006). Ultra-efficient replication of infectious prions by automated protein misfolding cyclic amplification. J. Biol. Chem. 281, 35245–35252. doi: 10.1074/jbc.M603964200 16982620

[B89] Salta E. Panagiotidis C. Teliousis K. Petrakis S. Eleftheriadis E. Arapoglou F. . (2009). Evaluation of the possible transmission of BSE and scrapie to gilthead sea bream (*Sparus aurata*). PLoS ONE 4:e6175. pone.0006175. doi: 10.1371/journal.pone.0006175 PMC2712096 19636413

[B90] Schreuder B. E. Geertsma R. E. van Keulen L. J. van Asten J. A. Enthoven P. Oberthür R. C. . (1998). Studies on the efficacy of hyperbaric rendering procedures in inactivating bovine spongiform encephalopathy (BSE) and scrapie agents. Vet. Rec. 142, 474–480. vr.142.18.474. doi: 10.1136/vr.142.18.474 9612912

[B91] Sohn H. J. Lee Y,.H Green R,.B Spencer Y,.I Hawkins S,.A. C Stack M,.J. . (2009). Bone marrow infectivity in cattle exposed to the bovine spongiform encephalopathy agent. Vet. Rec. 164, 272–273. doi: 10.1136/vr.164.9.272 19252214

[B92] Sørensen M. (2012). A review of the effects of ingredient composition and processing conditions on the physical qualities of extruded high-energy fish feed as measured by prevailing methods. Aquac. Nutr. 18, 233–248. doi: 10.1111/j.1365-2095.2011.00924.x

[B93] Spranghers T. (2017). Rearing of the black soldier fly towards application in piglet feed. (Ph.D. Thesis), Ghent, Belgium: Ghent University

[B94] STV (2024). Hoeveel Wordt er Verspild in Supermarkten? Infographic Retailcijfers 2023. Samen Tegen Voedselverspilling. Available online at: https://samentegenvoedselverspilling.nl/kennisbank/kb-hoeveel-wordt-er-verspild-in-supermarkten. (Accessed on October 18, 2024)

[B95] Suardi S. Vimercati C. Casalone C. Gelmetti D. Corona C. Iulini B. . (2012). Infectivity in skeletal muscle of cattle with atypical bovine spongiform encephalopathy. PloS ONE, 7:e31449. pone.0031449. doi: 10.1371/journal.pone.0031449 PMC3283643 22363650

[B96] Taylor D. M. Woodgate S. L. (2003). Rendering practices and inactivation of transmissible spongiform encephalopathy agents. Rev-Off. Int. Epizoot. 22, 297–310. doi: 10.20506/rst.22.1.1400 12793787

[B97] Tegtmeier D. Hurka S. Klüber P. Brinkrolf K. Heise P. Vilcinskas A. (2021). Cottonseed press cake as a potential diet for industrially farmed black soldier fly larvae triggers adaptations of their bacterial and fungal gut microbiota. Front. Microbiol. 12:634503. doi: 10.3389/fmicb.2021.634503 PMC8039154 33854488

[B98] UNEP (2024). Food Waste Index Report 2024. Think Eat Save: Tracking Progress to Halve Global Food Waste. United Nations Environment Programme. Available online at: https://www.unep.org/resources/publication/food-waste-index-report-2024. (Accessed on December 4, 2024)

[B99] Van der Fels-Klerx H. J. Meijer N. Nijkamp M. M. Schmitt E. Van Loon J. J. A. (2020). Chemical food safety of using former foodstuffs for rearing black soldier fly larvae (*Hermetia illucens*) for feed and food use. J. Insects Food Feed 6, 475–488. doi: 10.3920/JIFF2020.0024

[B100] Van Lieshout L. Knüppe J. (2023). Voedselverspilling bij Consumenten Thuis in Nederland in 2022. Stichting Voedingscentrum Nederland, Den Haag. Available online at: https://mobiel.voedingscentrum.nl/Assets/Uploads/voedingscentrum/Documents/Professionals/Pers/Pers%20overig/Rapport%20Voedselverspilling%202022.pdf. (Accessed on October 18, 2024)

[B101] Vandeweyer D. De Smet J. Van Looveren N. Van Campenhout L. (2021). Biological contaminants in insects as food and feed. J. Insects Food Feed 7, 807–822. doi: 10.3920/JIFF2020.0060

[B102] Vion (2024). Corporate Social Responsibility (CSR) Report 2023. Available online at: https://www.vionfoodgroup.com/wp-content/uploads/2024/11/CSR-report-2023.pdf. (Accessed on June 30, 2026)

[B103] Wagner D. R. Kline H. C. Martin M. S. Alexander L. R. Grandin T. Edwards- Callaway L. N. (2019). The effects of bolt length on penetration hole characteristics, brain damage and specified-risk material dispersal in finished cattle stunned with a penetrating captive bolt stunner. Meat Sci. 155, 109–114. j.meatsci.2019.05.006. doi: 10.1016/j.meatsci.2019.05.006 31103942

[B104] Wells G. A. Spiropoulos J. Hawkins S. A. Ryder S. J. (2005). Pathogenesis of experimental bovine spongiform encephalopathy: preclinical infectivity in tonsil and observations on the distribution of lingual tonsil in slaughtered cattle. Vet. Record 156, 401–407. doi: 10.1136/vr.156.13.401 15816193

[B105] Wells G. A. H. Konold T. Arnold M. E. Austin A. R. Hawkins S. A. C. Stack M. . (2007). Bovine spongiform encephalopathy: the effect of oral exposure dose on attack rate and incubation period in cattle. J. Gen. Virol. 88, 1363–1373. doi: 10.1099/vir.0.82421-0 17374783

[B106] Wisniewski H. M. Sigurdarson S. Rubenstein R. Kascsak R. J. Carp R. I. (1996). Mites as vectors for scrapie. Lancet 347, 1114. doi: 10.1016/S0140-6736(96)90310-4 8602080

[B107] WOAH (2023). Guidelines for Targeted BSE Surveillance. World Organisation for Animal Health. Available online at: https://www.woah.org/app/uploads/2023/07/ang-book-bse-guidelines-07072023-final.pdf. (Accessed on December 11, 2024)

[B108] WOAH (2024a). Bovine Spongiform Encephalopathy. World Organisation for Animal Health. Available online at: https://www.woah.org/en/disease/bovine-spongiform-encephalopathy/. (Accessed on December 11, 2024)

[B109] WOAH (2024b). WAHIS: World Animal Health Information System. World Organisation for Animal Health. Available online at: https://wahis.woah.org/#/home. [Accessed on December 5, 2024].

[B110] WOAH (2026). United Kingdom - Bovine Spongiform Encephalopathy - Immediate Notification. Available online at: https://wahis.woah.org/#/in-review/7773. (Accessed on September 3, 2026)

[B111] Wynants E. Frooninckx L. Crauwels S. Verreth C. De Smet J. Sandrock C. . (2019). Assessing the microbiota of black soldier fly larvae (*Hermetia illucens*) reared on organic waste streams on four different locations at laboratory and large scale. Microb. Ecol. 77, 913–930. doi: 10.1007/s00248-018-1286-x 30430196

[B112] Yamamoto T. Tsutsui T. Nonaka T. Kobayashi S. Nishiguchi A. Yamane I. (2006). A quantitative assessment of the risk of exposure to bovine spongiform encephalopathy via meat-and-bone meal in Japan. Prevent. Vet. Med. 75, 221–238. doi: 10.1016/j.prevetmed.2006.03.003 16712985

