# Supplementary material for: Quantitative risk assessment of BSE transmission if insects are reared on residual streams containing animal proteins
Source: Front Microbiol. 2026 Oct 23;17:1943428. doi: 10.3389/fmicb.2026.1943428 (PMC13739672; doi:10.3389/fmicb.2026.1943428)
Supplement: Supplementary file 1 [file Table_1.docx]

Supplementary Material

Table 1. Weight (g) of insects and frass after a 7-day rearing period for black soldier fly larvae (BSFL) and a 21-day rearing period for yellow mealworm (YMW) if grown on former food from supermarkets (SM), kitchen food scraps from consumer households (KF), category 2 meat meal from animal rendering (MM), and chicken feed (CF). Source: Meijer et al., 2026; Hoek-van den Hil et al., in prep.

| Insect species | Substrate | Experiment | Weight insects (g) | Weight frass (g) |
| --- | --- | --- | --- | --- |
| YMW | SM | 1 | 731 | 1617 |
| YMW | SM | 2 | 777 | 1601 |
| YMW | SM | 3 | 741 | 1737 |
| YMW | SM | 4 | 725 | 1650 |
| YMW | SM | 5 | 739 | 1627 |
| YMW | KF | 1 | 941 | 1454 |
| YMW | KF | 2 | 940 | 1447 |
| YMW | KF | 3 | 906 | 1501 |
| YMW | KF | 4 | 979 | 1450 |
| YMW | KF | 5 | 955 | 1494 |
| YMW | MM | 1 | 1597 | 1685 |
| YMW | MM | 2 | 1699 | 1605 |
| YMW | MM | 3 | 1558 | 1711 |
| YMW | MM | 4 | 1589 | 1705 |
| YMW | MM | 5 | 1607 | 1689 |
| BSFL | CF | 1 | 1509 | 2837 |
| BSFL | CF | 2 | 1284 | 2908 |
| BSFL | CF | 3 | 1396 | 2735 |
| BSFL | CF | 4 | 1350 | 2744 |
| BSFL | CF | 5 | 1821 | 2707 |
| BSFL | SM | 1 | 1758 | 3946 |
| BSFL | SM | 2 | 1437 | 3486 |
| BSFL | SM | 3 | 1466 | 3805 |
| BSFL | SM | 4 | 1586 | 3813 |
| BSFL | SM | 5 | 1625 | 3755 |
| BSFL | KF | 1 | 927 | 3296 |
| BSFL | KF | 2 | 657 | 3207 |
| BSFL | KF | 3 | 725 | 3058 |
| BSFL | KF | 4 | 734 | 3231 |
| BSFL | KF | 5 | 894 | 3390 |

Table 2. Median, 2.5 percentile, 97.5 percentile and 99 percentile values of infectivity in substrate, exposure of cattle to BSE infectivity and probability of ≥ 1 new infection for each substrate-insect combination. SM: former food from supermarkets; KF: kitchen food scraps from consumer households; MM: category 2 meat meal; BSFL: black soldier fly larvae; YMW: yellow mealworm.

|  | BSFL on SM | | | | YMW on SM | | | |
| --- | --- | --- | --- | --- | --- | --- | --- | --- |
| Model output | Median | 2.5p | 97.5p | 99p | Median | 2.5p | 97.5p | 99p |
| Infectivity in substrate (CoID_50_) | 9.4E-02 | 1.9E-02 | 5.1E-01 | 7.5E-01 | 9.4E-02 | 1.9E-02 | 5.1E-01 | 7.5E-01 |
| Overall exposure (CoID_50_) | 3.2E-06 | 6.1E-07 | 1.8E-05 | 2.6E-05 | 3.3E-06 | 6.2E-07 | 1.9E-05 | 2.7E-05 |
| Exposure via feed (CoID_50_) | 2.1E-06 | 4.2E-07 | 1.1E-05 | 1.6E-05 | 2.2E-06 | 4.4E-07 | 1.2E-05 | 1.8E-05 |
| Exposure via grazing - manure | 8.1E-08 | 1.3E-08 | 6.0E-07 | 9.3E-07 | 8.6E-08 | 1.3E-08 | 6.2E-07 | 9.9E-07 |
| Exposure via grazing - frass | 6.4E-07 | 1.0E-07 | 4.8E-06 | 7.5E-06 | 6.3E-07 | 9.8E-08 | 4.7E-06 | 7.2E-06 |
| Exposure via calf milk replacer | 1.3E-07 | 0.0E+00 | 2.6E-06 | 4.1E-06 | 1.4E-07 | 0.0E+00 | 2.7E-06 | 4.6E-06 |
| Overall probability >= 1 new BSE infection | 2.2E-06 | 4.3E-07 | 1.3E-05 | 1.8E-05 | 2.3E-06 | 4.3E-07 | 1.3E-05 | 1.9E-05 |
| Probability >= 1 new BSE infection via feed | 1.4E-06 | 2.9E-07 | 8.0E-06 | 1.1E-05 | 1.5E-06 | 3.1E-07 | 8.3E-06 | 1.2E-05 |
| Probability >= 1 new BSE infection via grazing - manure | 5.6E-08 | 8.8E-09 | 4.2E-07 | 6.4E-07 | 5.9E-08 | 9.3E-09 | 4.3E-07 | 6.9E-07 |
| Probability >= 1 new BSE infection via grazing - frass | 4.5E-07 | 7.0E-08 | 3.3E-06 | 5.2E-06 | 4.4E-07 | 6.8E-08 | 3.3E-06 | 5.0E-06 |
| Probability >= 1 new BSE infection via calf milk replacer | 8.8E-08 | 0.0E+00 | 1.8E-06 | 2.8E-06 | 9.4E-08 | 0.0E+00 | 1.9E-06 | 3.2E-06 |

Table 2. Continued.

|  | BSFL on KF | | | | YMW on KF | | | |
| --- | --- | --- | --- | --- | --- | --- | --- | --- |
| Model output | Median | 2.5p | 97.5p | 99p | Median | 2.5p | 97.5p | 99p |
| Infectivity in substrate (CoID_50_) | 1.3E-01 | 2.5E-02 | 7.0E-01 | 1.0E+00 | 1.3E-01 | 2.5E-02 | 7.0E-01 | 1.0E+00 |
| Overall exposure (CoID_50_) | 3.3E-06 | 6.1E-07 | 1.9E-05 | 2.8E-05 | 5.2E-06 | 1.0E-06 | 3.0E-05 | 4.3E-05 |
| Exposure via feed (CoID_50_) | 1.9E-06 | 3.7E-07 | 1.0E-05 | 1.5E-05 | 3.7E-06 | 7.5E-07 | 2.0E-05 | 3.0E-05 |
| Exposure via grazing - manure | 7.2E-08 | 1.1E-08 | 5.4E-07 | 8.6E-07 | 1.5E-07 | 2.3E-08 | 1.1E-06 | 1.7E-06 |
| Exposure via grazing - frass | 9.9E-07 | 1.5E-07 | 7.4E-06 | 1.1E-05 | 7.5E-07 | 1.2E-07 | 5.5E-06 | 8.6E-06 |
| Exposure via calf milk replacer | 1.1E-07 | 0.0E+00 | 2.3E-06 | 3.8E-06 | 2.3E-07 | 0.0E+00 | 4.6E-06 | 7.8E-06 |
| Overall probability >= 1 new BSE infection | 2.3E-06 | 4.2E-07 | 1.3E-05 | 1.9E-05 | 3.6E-06 | 7.0E-07 | 2.1E-05 | 3.0E-05 |
| Probability >= 1 new BSE infection via feed | 1.3E-06 | 2.6E-07 | 7.1E-06 | 1.0E-05 | 2.6E-06 | 5.2E-07 | 1.4E-05 | 2.1E-05 |
| Probability >= 1 new BSE infection via grazing - manure | 5.0E-08 | 7.8E-09 | 3.8E-07 | 6.0E-07 | 1.0E-07 | 1.6E-08 | 7.5E-07 | 1.2E-06 |
| Probability >= 1 new BSE infection via grazing - frass | 6.9E-07 | 1.1E-07 | 5.1E-06 | 7.9E-06 | 5.2E-07 | 8.1E-08 | 3.8E-06 | 6.0E-06 |
| Probability >= 1 new BSE infection via calf milk replacer | 7.9E-08 | 0.0E+00 | 1.6E-06 | 2.6E-06 | 1.6E-07 | 0.0E+00 | 3.2E-06 | 5.4E-06 |

Table 2. Continued.

|  | BSFL on MM | | | | YMW on MM | | | |
| --- | --- | --- | --- | --- | --- | --- | --- | --- |
| Model output | Median | 2.5p | 97.5p | 99p | Median | 2.5p | 97.5p | 99p |
| Infectivity in substrate (CoID_50_) | 4.2E-03 | 5.0E-04 | 4.7E-02 | 7.3E-02 | 4.2E-03 | 5.0E-04 | 4.7E-02 | 7.3E-02 |
| Overall exposure (CoID_50_) | 4.7E-08 | 4.6E-09 | 6.5E-07 | 1.1E-06 | 2.0E-07 | 2.4E-08 | 2.3E-06 | 3.7E-06 |
| Exposure via feed (CoID_50_) | 5.4E-09 | 6.4E-10 | 6.0E-08 | 9.6E-08 | 1.5E-07 | 1.9E-08 | 1.7E-06 | 2.7E-06 |
| Exposure via grazing - manure | 2.1E-10 | 1.9E-11 | 3.1E-09 | 5.2E-09 | 6.0E-09 | 5.5E-10 | 8.7E-08 | 1.5E-07 |
| Exposure via grazing - frass | 4.0E-08 | 3.7E-09 | 5.9E-07 | 9.6E-07 | 2.1E-08 | 1.9E-09 | 3.0E-07 | 4.9E-07 |
| Exposure via calf milk replacer | 3.0E-10 | 0.0E+00 | 1.1E-08 | 2.0E-08 | 8.8E-09 | 0.0E+00 | 3.2E-07 | 5.9E-07 |
| Overall probability >= 1 new BSE infection | 3.2E-08 | 3.2E-09 | 4.5E-07 | 7.4E-07 | 1.4E-07 | 1.6E-08 | 1.6E-06 | 2.6E-06 |
| Probability >= 1 new BSE infection via feed | 3.8E-09 | 4.5E-10 | 4.2E-08 | 6.7E-08 | 1.1E-07 | 1.3E-08 | 1.2E-06 | 1.9E-06 |
| Probability >= 1 new BSE infection via grazing - manure | 1.4E-10 | 1.3E-11 | 2.2E-09 | 3.6E-09 | 4.1E-09 | 3.8E-10 | 6.0E-08 | 1.0E-07 |
| Probability >= 1 new BSE infection via grazing - frass | 2.8E-08 | 2.6E-09 | 4.1E-07 | 6.7E-07 | 1.4E-08 | 1.3E-09 | 2.1E-07 | 3.4E-07 |
| Probability >= 1 new BSE infection via calf milk replacer | 2.1E-10 | 0.0E+00 | 7.9E-09 | 1.4E-08 | 6.1E-09 | 0.0E+00 | 2.3E-07 | 4.1E-07 |

Table 3. Median, 2.5 percentile, 97.5 percentile and 99 percentile values of the overall exposure (log_10_ CoID_50_) of cattle to BSE infectivity per substrate-insect combination for each of the what-if scenarios. SM: former food from supermarkets; KF: kitchen food scraps from consumer households; MM: category 2 meat meal; BSFL: black soldier fly larvae; YMW: yellow mealworm.

|  | BSFL on SM | | | | YMW on SM | | | |
| --- | --- | --- | --- | --- | --- | --- | --- | --- |
| Scenario | Median | 2.5p | 97.5p | 99p | Median | 2.5p | 97.5p | 99p |
| Baseline | 3.2E-06 | 6.1E-07 | 1.8E-05 | 2.6E-05 | 3.3E-06 | 6.2E-07 | 1.9E-05 | 2.7E-05 |
| WI-1 | 3.7E-06 | 6.8E-07 | 2.0E-05 | 2.8E-05 | 3.8E-06 | 7.1E-07 | 2.1E-05 | 2.9E-05 |
| WI-2 | 3.1E-06 | 5.7E-07 | 1.8E-05 | 2.5E-05 | 3.2E-06 | 6.0E-07 | 1.8E-05 | 2.6E-05 |
| WI-3 | 4.6E-05 | 8.8E-06 | 2.4E-04 | 3.2E-04 | 4.8E-05 | 9.2E-06 | 2.5E-04 | 3.3E-04 |
| WI-4 | 2.9E-08 | 1.7E-08 | 2.7E-07 | 2.4E-06 | 3.0E-08 | 1.8E-08 | 2.8E-07 | 2.4E-06 |
| WI-5 | 1.1E-03 | 2.1E-04 | 5.9E-03 | 7.8E-03 | 1.2E-03 | 2.1E-04 | 6.1E-03 | 8.2E-03 |
| WI-6 | 8.2E-06 | 1.6E-06 | 4.6E-05 | 6.4E-05 | 8.3E-06 | 1.6E-06 | 4.7E-05 | 6.4E-05 |
| WI-7 | 9.1E-07 | 1.3E-07 | 6.5E-06 | 9.5E-06 | 9.1E-07 | 1.3E-07 | 6.5E-06 | 9.5E-06 |
| WI-8 | 1.5E-06 | 2.6E-07 | 9.2E-06 | 1.3E-05 | 1.5E-06 | 2.7E-07 | 9.2E-06 | 1.3E-05 |
| WI-9 | 1.9E-06 | 3.3E-07 | 1.1E-05 | 1.6E-05 | 2.0E-06 | 3.4E-07 | 1.2E-05 | 1.6E-05 |
| WI-10 | 1.4E-06 | 2.4E-07 | 9.2E-06 | 1.3E-05 | 1.4E-06 | 2.5E-07 | 9.3E-06 | 1.4E-05 |
| WI-11 | 4.3E-05 | 8.4E-06 | 2.4E-04 | 3.2E-04 | 4.5E-05 | 8.9E-06 | 2.5E-04 | 3.4E-04 |
| WI-12 | 2.1E-03 | 4.1E-04 | 1.1E-02 | 1.6E-02 | 2.2E-03 | 4.3E-04 | 1.2E-02 | 1.6E-02 |
| WI-13 | 5.8E-06 | 1.0E-06 | 3.6E-05 | 5.1E-05 | 5.9E-06 | 1.0E-06 | 3.5E-05 | 5.1E-05 |
| WI-14 | 3.3E-06 | 6.3E-07 | 1.8E-05 | 2.6E-05 | 3.4E-06 | 6.5E-07 | 1.9E-05 | 2.7E-05 |
| WI-15 | 3.7E-06 | 6.9E-07 | 2.1E-05 | 3.0E-05 | 3.8E-06 | 7.1E-07 | 2.2E-05 | 3.0E-05 |
| WI-16 | 3.1E-06 | 6.0E-07 | 1.7E-05 | 2.4E-05 | 3.2E-06 | 6.2E-07 | 1.8E-05 | 2.5E-05 |
| WI-17 | 4.8E-05 | 8.7E-06 | 2.7E-04 | 3.8E-04 | 4.9E-05 | 8.9E-06 | 2.8E-04 | 3.9E-04 |
| WI-18 | 7.1E-05 | 1.1E-05 | 4.6E-04 | 6.6E-04 | 7.6E-05 | 1.2E-05 | 4.8E-04 | 6.7E-04 |
| WI-19 | 2.9E-06 | 5.5E-07 | 1.6E-05 | 2.3E-05 | 3.0E-06 | 5.7E-07 | 1.6E-05 | 2.3E-05 |
| WI-20 | 4.0E-06 | 7.0E-07 | 2.6E-05 | 3.8E-05 | 4.1E-06 | 7.3E-07 | 2.7E-05 | 4.0E-05 |
| WI-21 | 1.2E-05 | 2.1E-07 | 2.2E-04 | 3.3E-04 | 1.3E-05 | 2.0E-07 | 2.3E-04 | 3.5E-04 |

Table 3. Continued.

|  | BSFL on KF | | | | YMW on KF | | | |
| --- | --- | --- | --- | --- | --- | --- | --- | --- |
| Scenario | Median | 2.5p | 97.5p | 99p | Median | 2.5p | 97.5p | 99p |
| Baseline | 3.3E-06 | 6.1E-07 | 1.9E-05 | 2.8E-05 | 5.2E-06 | 1.0E-06 | 3.0E-05 | 4.3E-05 |
| WI-1 | 3.8E-06 | 6.9E-07 | 2.1E-05 | 2.9E-05 | 6.1E-06 | 1.1E-06 | 3.3E-05 | 4.5E-05 |
| WI-2 | 3.2E-06 | 5.8E-07 | 1.9E-05 | 2.6E-05 | 5.2E-06 | 9.6E-07 | 2.9E-05 | 4.0E-05 |
| WI-3 | 4.8E-05 | 9.0E-06 | 2.5E-04 | 3.5E-04 | 7.6E-05 | 1.5E-05 | 3.9E-04 | 5.2E-04 |
| WI-4 | 3.0E-08 | 1.7E-08 | 2.7E-07 | 2.3E-06 | 4.9E-08 | 2.8E-08 | 4.4E-07 | 3.9E-06 |
| WI-5 | 1.2E-03 | 2.1E-04 | 6.2E-03 | 8.4E-03 | 1.8E-03 | 3.4E-04 | 9.6E-03 | 1.3E-02 |
| WI-6 | 1.1E-05 | 2.2E-06 | 6.2E-05 | 8.6E-05 | 1.1E-05 | 2.2E-06 | 6.3E-05 | 8.7E-05 |
| WI-7 | 1.2E-06 | 1.8E-07 | 8.8E-06 | 1.3E-05 | 1.2E-06 | 1.8E-07 | 8.8E-06 | 1.3E-05 |
| WI-8 | 1.8E-06 | 2.9E-07 | 1.1E-05 | 1.6E-05 | 2.3E-06 | 4.0E-07 | 1.4E-05 | 1.9E-05 |
| WI-9 | 2.2E-06 | 3.7E-07 | 1.3E-05 | 1.9E-05 | 3.0E-06 | 5.2E-07 | 1.8E-05 | 2.5E-05 |
| WI-10 | 1.7E-06 | 2.8E-07 | 1.1E-05 | 1.6E-05 | 2.1E-06 | 3.6E-07 | 1.3E-05 | 2.0E-05 |
| WI-11 | 3.9E-05 | 7.5E-06 | 2.1E-04 | 2.9E-04 | 7.7E-05 | 1.5E-05 | 4.2E-04 | 5.8E-04 |
| WI-12 | 1.9E-03 | 3.6E-04 | 1.0E-02 | 1.4E-02 | 3.8E-03 | 7.3E-04 | 2.0E-02 | 2.8E-02 |
| WI-13 | 7.3E-06 | 1.2E-06 | 4.6E-05 | 6.6E-05 | 8.4E-06 | 1.5E-06 | 5.0E-05 | 7.0E-05 |
| WI-14 | 3.4E-06 | 6.2E-07 | 1.9E-05 | 2.7E-05 | 5.4E-06 | 1.0E-06 | 3.1E-05 | 4.3E-05 |
| WI-15 | 4.1E-06 | 7.2E-07 | 2.4E-05 | 3.4E-05 | 5.9E-06 | 1.1E-06 | 3.4E-05 | 4.7E-05 |
| WI-16 | 3.2E-06 | 6.0E-07 | 1.8E-05 | 2.6E-05 | 5.1E-06 | 1.0E-06 | 2.9E-05 | 4.0E-05 |
| WI-17 | 5.1E-05 | 9.0E-06 | 3.0E-04 | 4.3E-04 | 7.6E-05 | 1.4E-05 | 4.3E-04 | 6.2E-04 |
| WI-18 | 6.4E-05 | 1.0E-05 | 4.1E-04 | 5.9E-04 | 1.3E-04 | 2.0E-05 | 8.1E-04 | 1.2E-03 |
| WI-19 | 3.0E-06 | 5.5E-07 | 1.7E-05 | 2.4E-05 | 4.7E-06 | 9.1E-07 | 2.6E-05 | 3.7E-05 |
| WI-20 | 4.0E-06 | 7.0E-07 | 2.6E-05 | 3.7E-05 | 6.7E-06 | 1.2E-06 | 4.4E-05 | 6.5E-05 |
| WI-21 | 1.2E-05 | 3.2E-07 | 1.9E-04 | 3.0E-04 | 2.2E-05 | 2.5E-07 | 3.9E-04 | 5.9E-04 |

Table 3. Continued.

|  | BSFL on MM | | | | YMW on MM | | | |
| --- | --- | --- | --- | --- | --- | --- | --- | --- |
| Scenario | Median | 2.5p | 97.5p | 99p | Median | 2.5p | 97.5p | 99p |
| Baseline | 4.7E-08 | 4.6E-09 | 6.5E-07 | 1.1E-06 | 2.0E-07 | 2.4E-08 | 2.3E-06 | 3.7E-06 |
| WI-1 | 5.9E-08 | 5.1E-09 | 7.7E-07 | 1.2E-06 | 2.6E-07 | 2.7E-08 | 2.8E-06 | 4.2E-06 |
| WI-2 | 4.7E-08 | 4.1E-09 | 6.3E-07 | 9.4E-07 | 2.0E-07 | 2.1E-08 | 2.3E-06 | 3.5E-06 |
| WI-3 | 6.9E-07 | 6.2E-08 | 9.0E-06 | 1.3E-05 | 3.0E-06 | 3.2E-07 | 3.3E-05 | 4.8E-05 |
| WI-4 | 7.2E-10 | 1.2E-10 | 7.5E-09 | 1.2E-08 | 3.1E-09 | 6.6E-10 | 2.7E-08 | 4.5E-08 |
| WI-5 | 1.7E-05 | 1.5E-06 | 2.2E-04 | 3.2E-04 | 7.3E-05 | 7.5E-06 | 8.0E-04 | 1.2E-03 |
| WI-6 | 3.7E-07 | 4.1E-08 | 4.2E-06 | 6.4E-06 | 3.7E-07 | 4.0E-08 | 4.2E-06 | 6.3E-06 |
| WI-7 | 4.1E-08 | 3.4E-09 | 5.8E-07 | 8.7E-07 | 4.1E-08 | 3.4E-09 | 5.8E-07 | 8.7E-07 |
| WI-8 | 4.2E-08 | 3.6E-09 | 5.9E-07 | 8.9E-07 | 8.5E-08 | 8.6E-09 | 1.0E-06 | 1.5E-06 |
| WI-9 | 4.4E-08 | 3.9E-09 | 6.1E-07 | 9.0E-07 | 1.1E-07 | 1.1E-08 | 1.3E-06 | 2.1E-06 |
| WI-10 | 4.3E-08 | 3.6E-09 | 5.9E-07 | 8.9E-07 | 7.9E-08 | 7.7E-09 | 9.7E-07 | 1.4E-06 |
| WI-11 | 1.6E-07 | 1.6E-08 | 1.8E-06 | 2.7E-06 | 3.2E-06 | 3.5E-07 | 3.5E-05 | 5.6E-05 |
| WI-12 | 5.5E-06 | 6.1E-07 | 6.2E-05 | 9.6E-05 | 1.6E-04 | 1.7E-05 | 1.7E-03 | 2.7E-03 |
| WI-13 | 2.1E-07 | 1.8E-08 | 2.9E-06 | 4.4E-06 | 3.0E-07 | 3.1E-08 | 3.5E-06 | 5.1E-06 |
| WI-14 | 4.8E-08 | 4.3E-09 | 6.4E-07 | 9.6E-07 | 2.2E-07 | 2.3E-08 | 2.5E-06 | 3.7E-06 |
| WI-15 | 7.8E-08 | 6.8E-09 | 1.1E-06 | 1.6E-06 | 2.3E-07 | 2.4E-08 | 2.6E-06 | 3.9E-06 |
| WI-16 | 4.7E-08 | 4.3E-09 | 6.3E-07 | 9.5E-07 | 2.0E-07 | 2.2E-08 | 2.3E-06 | 3.4E-06 |
| WI-17 | 9.0E-07 | 7.9E-08 | 1.2E-05 | 1.8E-05 | 3.0E-06 | 3.2E-07 | 3.4E-05 | 5.1E-05 |
| WI-18 | 2.3E-07 | 2.3E-08 | 3.0E-06 | 4.6E-06 | 5.4E-06 | 4.9E-07 | 6.8E-05 | 1.1E-04 |
| WI-19 | 4.6E-08 | 4.2E-09 | 6.3E-07 | 9.4E-07 | 1.8E-07 | 2.0E-08 | 2.1E-06 | 3.2E-06 |
| WI-20 | 5.1E-08 | 4.6E-09 | 6.6E-07 | 1.0E-06 | 2.7E-07 | 2.6E-08 | 3.4E-06 | 5.1E-06 |
| WI-21 | 8.7E-08 | 6.0E-09 | 1.3E-06 | 2.1E-06 | 8.2E-07 | 4.8E-09 | 2.6E-05 | 4.2E-05 |

Figure 1. Relative contribution of the exposure pathways to the overall exposure of cattle to BSE infectivity in the baseline scenario and 21 what-if scenarios for the different substrate-insect combinations. Feed: exposure via cross-contamination of ruminant feed; Manure: exposure via grass or forage crops fertilized with pig or poultry manure; Frass: exposure via grass or forage crops fertilized with frass; CMR: exposure via calf milk replacer. A detailed description of the what-if scenarios is given in Table 2 of the manuscript.

Figure 2. Median values and 95% uncertainty interval of the estimated BSE risk of the different substrate-insect combinations if all adverse conditions explored in the what-if analysis would occur simultaneously (scenarios WI-5, WI-6, WI-12, WI-13, WI-14, WI-18 and WI-20 combined). A) Estimated overall exposure (log_10_ CoID_50_) of cattle to BSE infectivity. B) Estimated probability (log_10_ value) of ≥ 1 new BSE infection. SM: former food from supermarkets; KF: kitchen food scraps from consumer households; MM: category 2 meat meal; BSFL: black soldier fly larvae; YMW: yellow mealworm.
